# A Highly Efficient and Reusable Palladium(II)/Cationic 2,2’-Bipyridyl-Catalyzed Stille Coupling in Water

**DOI:** 10.3390/molecules21091205

**Published:** 2016-09-09

**Authors:** Wei-Yi Wu, Ling-Jun Liu, Fen-Ping Chang, Yu-Lun Cheng, Fu-Yu Tsai

**Affiliations:** Institute of Organic and Polymeric Materials, National Taipei University of Technology, 1, Sec. 3, Chung-Hsiao E. Rd., Taipei 10608, Taiwan; willy.wu@lcygroup.com (W.-Y.W.); lingebubest@gmail.com (L.-J.L.); a49001103@yahoo.com.tw (F.-P.C.); jacky0205333@yahoo.com.tw (Y.-L.C.)

**Keywords:** stille coupling, water-soluble catalyst, reusable, aryl halides, organostannane

## Abstract

A water-soluble PdCl_2_(NH_3_)_2_/cationic 2,2′-bipyridyl system was found to be a highly efficient catalyst for Stille coupling of aryl iodides and bromides with organostannanes. The coupling reaction was conducted at 110 °C in water, under aerobic conditions, in the presence of NaHCO_3_ as a base to afford corresponding Stille coupling products in good to high yields. When aryltributylstannanes were employed, the reactions proceeded smoothly under a very low catalyst loading (as little as 0.0001 mol %). After simple extraction, the residual aqueous phase could be reused in subsequent runs, making this Stille coupling economical. In the case of tetramethylstannane, however, a greater catalyst loading (1 mol %) and the use of tetraethylammonium iodide as a phase-transfer agent were required in order to obtain satisfactory yields.

## 1. Introduction

The palladium-catalyzed cross-coupling of aryl halides or pseudo-halides with organo-stannanes, known as the Stille coupling, is one of the most powerful methods for the straightforward construction of carbon–carbon bonds in synthetic chemistry [1,2,3]. The main advantages of the Stille coupling reaction include the stability and functional group tolerance of stannanes, the broad reaction scope of aryl halides and pseudo-halides, and its chemoselectivity; therefore, this reaction has been widely applied in natural product synthesis [4,5,6,7,8,9,10], biological research [11], and for pharmaceutical purposes [12]. Stille coupling reactions are generally carried out in organic solvents under homogeneous catalysis, and, hence, it is difficult to separate the catalyst from the reaction mixture and then recycle it at the end of the reaction, leading to wastage of precious metals. Therefore, the development of a recyclable and reusable catalytic system is highly attractive and valuable from the viewpoints of green chemistry and practical application. To circumvent this problem, several new strategies involving heterogenized homogeneous catalysts have been developed for recycling and reusing catalysts, including the use of Pd complexes supported by silica gel [13,14], polymers [15,16], nanoparticles [17,18], porous metal−organic frameworks [19], bulky proazaphosphatrane ligands [20], mesoporous silica [21,22,23,24,25,26], and metal nanoparticles [27,28,29,30,31,32,33,34,35,36,37,38,39,40,41,42]. Alternatively, combinations of palladium complexes with several green solvents, such as ionic liquids [43,44,45], polyethylene glycol [46,47,48,49,50], H_2_O [51,52,53,54], or H_2_O in the presence of surfactants [55,56,57], have also been applied as reusable catalytic systems.

**Scheme 1:**
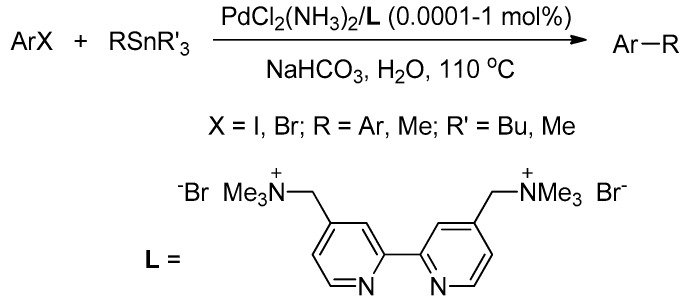
Palladium-catalyzed Stille coupling in water.

We recently reported that cationic 2,2′-bipyridyl is an excellent ligand to bring PdCl_2_(NH_3_)_2_ into the aqueous phase, in order to efficiently perform several carbon–carbon bond-formation reactions, and the residual aqueous solution can be reused for the next run [58,59,60,61,62,63]. As part of our continuing efforts in the development of water-soluble and reusable catalytic systems for carbon–carbon bond-forming reactions, we report, herein, on a reusable PdCl_2_(NH_3_)_2_/cationic 2,2′-bipyridyl catalytic system, which can be applied for Stille coupling of aryl halides with organostannanes in water under aerobic conditions, in the presence of NaHCO_3_ as a base. The loading amount of the catalyst in a single batch reaction could be reduced to as little as 0.0001 mol % (1 ppm), still affording products in high yields (Scheme 1).

## 2. Results and Discussion

### 2.1. Optimization of Stille Coupling Conditions

Water-soluble cationic 2,2′-bipyridyl ligand **L** was synthesized according to our previously-published method [58,59]. The catalytic system was prepared by mixing equimolar amounts of PdCl_2_(NH_3_)_2_ and **L** in water, and then it was stored under air. Stock solutions of this catalytic system were prepared at different concentrations to obtain various catalyst loadings. In order to discover the optimal conditions, the coupling of 4′-iodoacetophenone (**1a**, 1 mmol) and PhSnBu_3_ (**2a**, 1.2 mmol) in the presence of PdCl_2_(NH_3_)_2_/**L** (1 mol %) in water (3 mL), at 110 °C for 0.5 h, was first investigated, and the results are summarized in Table 1.

Initially, several commonly-used bases were screened, and it was found that the use of NaHCO_3_ provided the Stille coupling product in a 95% yield, which was higher than the yields obtained using other inorganic bases (Entries 1–6). Then, two additional experiments were performed to demonstrate the necessity of water-soluble ligand **L**. Under the same conditions as Entry 6, only a 32% yield of the cross-coupling product was obtained in the absence of the ligand (Entry 7), and a 35% yield was obtained when **L** was replaced with neutral 2,2′-bipydryl (Entry 8). These results clearly revealed that use of the water-soluble ligand was crucial in this Stille coupling reaction. When basic aqueous soluble 2,2′-bipyridine-4,4′-dicarboxylic acid was employed as ligand, however, Stille coupling did not occur, hence, **1a** and **2a** remained intact (Entry 9). Other phenylstannane sources, such as PhSnCl_3_ and PhSnMe_3_, were also examined. Although these two reagents furnished **3aa** in 90% and 95% yields, respectively (Entries 10 and 11), PhSnBu_3_ can be synthesized from the much cheaper ClSnBu_3_; hence, aryltributylstannanes were applied for the reactions.

### 2.2. Reuse Studies of the Residual Aqueous Solution

**Scheme 2:**
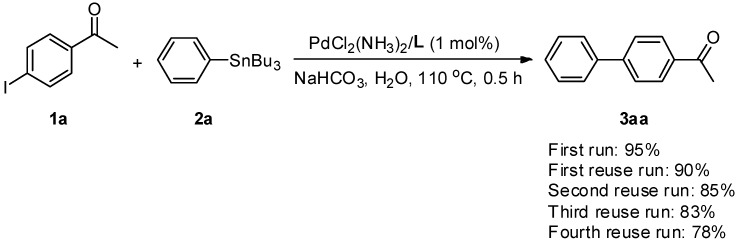
Catalyst reuse studies in Stille coupling of **1a** and **2a**.

We then studied the reusability of the aqueous catalytic system for Stille coupling, which is important from the viewpoints of practical utilization and economics. Coupling of **1a** and **2a** under the conditions of Entry 6, in Table 1, was performed in order to test the feasibility of this approach (Scheme 2). After completion of the first run, the organic portion was easily separated from the aqueous phase by simple extraction with hexane (3 mL × 3), and **3aa** was isolated in a 95% yield using a typical work-up procedure. The residual aqueous solution was then subjected to the next reaction run, charged with the same reactants, **1a** and **2a**, and NaHCO_3_. It was found that this residual aqueous solution could be reused at least four times, and a 78% isolated yield was reached in the fourth reuse run. In order to know the partitioning of the catalyst in the organic phase, the first run was performed again. After extracting the reaction mixture with hexane, the organic phase was then used for ICP-MASS analysis. It was found that there was no leaching of Pd into the organic phase. Thus, the slight decrease in activity was presumably due to a gradual decay of the catalytic activity.

### 2.3. Scope of Substrates and Loading Amounts of Catalyst

Encouraged by the excellent results of the reuse studies of the residual aqueous solution, we then examined the scope of substrates, and attempted to reduce the catalyst loading required (Table 2). Aryl iodides **1a** and **1b** with electron-withdrawing groups at the *para*-position coupled with various aryltributylstannanes **2a**–**2c** under 0.01 mol % catalyst loading, giving the corresponding Stille coupling products at yields between 90% and 98%, in 3 h (Entries 1, 3 and 4, 6, and 8 and 9). Further reduction of the catalyst loading to 0.0001 mol % (1 ppm), and increase of the reaction scale to 10 mmol, resulted in the corresponding Stille coupling products being obtained at yields between 72% and 82%, in 48 h (Entries 2, 5, and 7), and the highest turnover number (TON) achieved was up to 820,000 (Entry 2). Iodobenzene (**1c**) showed only a slightly lower rate compared with electron-withdrawing **1a** and **1b**, but still resulted in excellent yields by prolonging the reaction time to 6 h (Entries 10–12). For aryl iodides bearing an electron-donating group at the *para*-position, **1d** and **1e**, high yields were isolated in 6 h, with a catalyst loading of 0.01 mol % (Entries 13, 15–17, and 19 and 20). Similarly, in the cases of entries 14 and 18, 59% and 66% yields were obtained, respectively, in 48 h, under 1 ppm catalyst loading, using a reaction scale of 10 mmol (Entries 14 and 18).

Analogous reactions of cheaper aryl bromides were also investigated (Table 3). Activated aryl bromides **4a** and **4b**, were efficiently coupled with **2a**–**c** under conditions identical to those used for aryl iodides; however, a longer reaction time was required (Entries 1–9).

A very low catalyst loading could also be applied when employing electron-withdrawing aryl bromides. For example, the coupling of **4a** and **2a** furnished **3aa** in a 70% yield with a 1 ppm catalyst loading in 48 h (Entry 2). In the cases of **4c** and electron-donating **4e**, the reactions were much slower than those of the iodide analogs. Hence, conduction of the reaction using a 1 mol % catalyst loading and prolongation of the reaction time were necessary in order to obtain satisfactory yields (Entries 10–15). These results indicated that the oxidative addition of a carbon–bromine bond to palladium may be the rate-determining-step in this catalytic cycle. Surprisingly, the reaction rate was dramatically enhanced when deactivated 4-bromophenol (**4f**) was employed (Entries 16–20). Compound **4f** was soluble in basic aqueous solution, producing 4-bromophenoxide. This aryl bromide then underwent oxidative addition to palladium under homogeneous conditions, making this step much faster than for other water-insoluble aryl bromides. Taking advantage of this water-soluble property, **4f** coupled with **2a** very smoothly, providing a 56% yield (TON = 560,000) of **3fa** under a catalyst loading of only 1 ppm at 110 °C for 48 h (Entry 18).

The utility of this reaction protocol for the formation of C*sp*^2^–C*sp*^3^ carbon–carbon bonds was also evaluated. As illustrated in Table 4, the coupling of **1a** and SnMe_4_, **5**, using 1 mol % catalyst loading at 110 °C for 24 h, gave **6a** in only a 51% yield (Entry 1). The use of two equivalents of **5** in the reaction was owing to its low boiling point (74–75 °C). In order to improve upon this outcome, a phase-transfer agent was added into the reaction [55,56,57]. The use of tetrabutylammonium bromide (TBAB) and tetrabutylammonium hydroxide (TBAOH) led to the formation of **6a** in yields of 63% and 70%, respectively (Entries 2 and 3). It is worth noting that a 91% isolated yield of **6a** could be achieved when tetraethylammonium iodide (TEAI) was applied in the reaction system (Entry 4). Thus, **1b** coupled with **5** under such conditions afforded **6b** in a 78% yield (Entry 5). However, a low product yield was obtained when electron-rich **1e** was utilized (Entry 6). Activated aryl bromides **4a** and **4b** were also coupled with **5**, furnishing **6a** and **6b** in 40% and 31% yields, respectively (Entries 7 and 8).

## 3. Experimental Section

### 3.1. General Information

Chemicals were purchased from commercial suppliers and were used without further purification. Cationic 2,2′-bipyridyl ligand was prepared according to published procedures [58,59]. Aryltributylstannanes were prepared according to known procedures [64]. All ^1^H- and ^13^C-NMR spectra were recorded in CDCl_3_ or DMSO-*d*_6_ at 25 °C on a Bruker Biospin AG 300 NMR spectrometer (Bruker Co., Faellanden, Switzerland), in which chemical shifts (δ in ppm) were determined with respect to the non-deuterated solvent as a reference (^1^H-NMR: CHCl_3_ at 7.24, non-deuterated DMSO at 2.49 ppm; ^13^C-NMR: CDCl_3_ at 77.0, DMSO-*d*_6_ at 39.5 ppm). Melting points were recorded using a melting point apparatus, and were uncorrected.

### 3.2. Typical Stille Coupling Procedure

A sealable tube, equipped with a magnetic stirring bar, was charged with aryl halide (1 mmol), organotin (1.2 mmol), NaHCO_3_ (2 mmol), and H_2_O (2 mL). In the case of tetramethyltin, the addition of tetraethylammonium iodide (TEAI, 1 mmol) was required. After the addition of PdCl_2_(NH_3_)_2_/**L** aqueous solution (1 mL H_2_O; different concentrations were required for various substrate/catalyst ratios), the tube was sealed under air using a Teflon-coated screw cap. The reaction vessel was then placed in an oil bath at 110 °C for the indicated reaction duration (see Table 2, Table 3 and Table 4). After cooling of the reaction mixture to room temperature, the aqueous solution was extracted with hexane or ethyl acetate; the organic phase was dried over MgSO_4_, and the solvent was then removed under vacuum. Column chromatography on silica gel afforded the desired product (see Supplementary Materials for the copies of NMR spectra).

*4-Phenylacetophenone* (**3aa**, Table 2, Entries 1 and 2, and Table 3, Entries 1 and 2). CAS: 92-91-1; white solid (m.p. = 119–121 °C, lit. [59] 119–121 °C). ^1^H-NMR (CDCl_3_): δ 2.62 (s, 3H), 7.39–7.41 (m, 1H), 7.44–7.48 (m, 2H), 7.60–7.63 (m, 2H), 7.66–7.68 (m, 2H), 8.01–8.03 (m, 2H); ^13^C-NMR (CDCl_3_): δ 26.6, 127.1(2C), 127.2 (2C), 128.2, 128.8 (2C), 128.9 (2C), 135.7, 139.8, 145.7, 197.7.

*4-Acetyl-4′-fluorobiphenyl* (**3ab**, Table 2, Entry 3, and Table 3, Entries 3 and 4). CAS: 720-74-1; white solid (m.p. = 103–104 °C, lit. [59] 103–104 °C). ^1^H-NMR (CDCl_3_): δ 2.62 (s, 3H), 7.14 (t, *J* = 9.0 Hz, 2H), 7.55–7.63 (m, 4H), 7.99–8.02 (m, 2H); ^13^C-NMR (CDCl_3_): δ 26.6, 115.9 (d, *J*_C-F_ = 22.5 Hz, 2C), 127.0 (2C), 128.8 (d, *J*_C-F_ = 7.5 Hz, 2C), 128.9 (2C), 135.8, 136.0 (d, *J*_C-F_ = 3.0 Hz), 144.7, 163.0 (d, *J*_C-F_ = 247.5 Hz), 197.7.

*4-Acetyl-4′-methoxybiphenyl* (**3ac**, Table 2, Entries 4 and 5, and Table 3, Entries 5 and 6). CAS: 13021-18-6; pale yellow solid (m.p. = 153–155 °C, lit. [59] 153–155 °C). ^1^H-NMR (CDCl_3_): δ 2.60 (s, 3H), 3.84 (s, 3H), 6.98 (d, *J* = 9.0 Hz, 2H), 7.56 (d, *J* = 9.0 Hz, 2H), 7.62 (d, *J* = 9.0 Hz, 2H), 7.99 (d, *J* = 9.0 Hz, 2H); ^13^C-NMR (CDCl_3_): δ 26.6, 55.3, 114.3 (2C), 126.5 (2C), 128.3 (2C), 128.9 (2C), 132.1, 135.2, 145.3, 159.8, 197.7.

*4-Phenylbenzonitrile* (**3ba**, Table 2, Entries 6 and 7, and Table 3, Entry 7). CAS: 2920-38-9; white solid (m.p. = 89–91 °C, lit. [65] 89–90 °C). ^1^H-NMR (CDCl_3_): δ 7.38–7.50 (m, 3H), 7.55–7.59 (m, 2H), 7.65–7.73 (m, 4H); ^13^C-NMR (CDCl_3_): δ 110.8, 118.9, 127.2 (2C), 127.7 (2C), 128.6, 129.1 (2C), 132.6 (2C), 139.1, 145.6.

*4-(4-Fluorophenyl)benzonitrile* (**3bb**, Table 2, Entry 8 and Table 3, Entry 8). CAS: 10540-31-5; white solid (m.p. = 115–117 °C, lit. [66] 116–118 °C). ^1^H-NMR (CDCl_3_): δ 7.15 (t, *J* = 9.0 Hz, 2H), 7.52–7.56 (m, 2H), 7.60–7.63 (m, 2H), 7.68–7.61 (m, 2H); ^13^C-NMR (CDCl_3_): δ 110.9, 115.9 (d, *J*_C-F_ = 21.8 Hz, 2C), 118.8, 127.5 (2C), 128.9 (d, *J*_C-F_ = 8.3 Hz, 2C), 132.6 (2C), 135.2 (d, *J*_C-F_ =3.0 Hz), 144.6, 163.1 (d, *J*_C-F_ = 246.8 Hz).

*4′-Methoxybiphenyl-4-carbonitrile* (**3bc**, Table 2, Entry 9 and Table 3, Entry 9). CAS: 58743-77-4; pale yellow solid (m.p. = 104–106 °C, lit. [67] 104–105 °C). ^1^H-NMR (CDCl_3_): δ 3.84 (s, 3H), 6.99 (d, *J* = 9.0 Hz, 2H), 7.52 (d, *J* = 9.0 Hz, 2H), 7.60–7.69 (m, 4H); ^13^C-NMR (CDCl_3_): δ 55.4, 100.4 114.5 (2C), 119.1, 127.1 (2C), 128.3 (2C), 131.4, 132.5 (2C), 145.2, 160.2.

*Biphenyl* (**3ca**, Table 2, Entry 10 and Table 3, Entry 10). CAS: 92-52-4; white solid (m.p. = 71–72 °C, lit [68] 71–72 °C). ^1^H-NMR (CDCl_3_): δ 7.34–7.40 (m, 2H), 7.44–7.49 (m, 4H), 7.61–7.64 (m, 4H); ^13^C-NMR (CDCl_3_): δ 127.1 (4C), 127.2(2C), 128.7 (4C), 141.2 (2C).

*4-Fluorobiphenyl* (**3cb**, Table 2, Entry 11 and Table 3, Entry 11). CAS: 324-74-3; white solid (m.p. = 72–73 °C, lit. [69] 72–73 °C). ^1^H-NMR (CDCl_3_): δ 7.10–7.15 (m, 2H), 7.34–7.37 (m,1H), 7.41–7.46 (m, 2H), 7.52–7.61 (m, 4H); ^13^C-NMR (CDCl_3_): δ 115.6 (d, *J*_C-F_ = 21.0 Hz, 2C), 127.0 (2C), 127.2, 128.7 (d, *J*_C-F_ = 8.3 Hz, 2C), 128.8 (2C), 137.3 (d, *J*_C-F_ = 3.0 Hz), 140.2, 162.4 (d, *J*_C-F_ = 245.3 Hz).

*4-Methoxybiphenyl* (**3cc**, Table 2, Entries 12, 17 and 18, and Table 3, Entries 12 and 13). CAS: 613-37-6; white solid (m.p. = 85–87 °C, lit. [59] 85–87 °C). ^1^H-NMR (CDCl_3_): δ 3.85 (s, 3H), 6.99 (d, *J* = 9.0 Hz, 2H), 7.31–7.34 (m, 1H), 7.40–7.45 (m, 2H), 7.52–7.58 (m, 4H); ^13^C-NMR (CDCl_3_): δ 55.3, 114.2 (2C), 126.6, 126.7 (2C), 128.1 (2C), 128.7 (2C), 133.7, 140.8, 159.1.

*4-Methylbiphenyl* (**3da**, Table 2, Entries 13 and 14). CAS: 644-08-6; white solid (m.p. = 43–45 °C, lit. [59] 43–45 °C). ^1^H-NMR (CDCl_3_): δ 2.41 (s, 3H), 7.24–7.28 (m, 2H), 7.34–7.36 (m, 1H), 7.42–7.47 (m, 2H), 7.50–7.53 (m, 2H), 7.58–7.61 (m, 2H); ^13^C-NMR (CDCl_3_): δ 21.1, 126.8, 126.9 (4C), 128.7 (2C), 129.5 (2C), 137.0, 138.3, 141.1.

*4-Fluoro-4′-methylbiphenyl* (**3db**, Table 2, Entry 15). CAS: 72093-43-7; white solid (m.p. = 79–81 °C, lit. [70] 78–79 °C). ^1^H-NMR (CDCl_3_): δ 2.39 (s, 3H), 7.11 (t, *J* = 9.0 Hz, 2H), 7.23–7.25 (m, 2H), 7.42–7.45 (m, 2H), 7.50–7.54 (m, 2H); ^13^C-NMR (CDCl_3_): δ 21.1, 115.6 (d, *J*_C-F_ = 21.8 Hz, 2C), 126.8 (2C), 128.5 (d, *J*_C-F_ = 8.3 Hz, 2C), 129.5 (2C), 137.0, 137.2 (d, *J*_C-F_ = 3.0 Hz), 137.4, 162.3 (d, *J*_C-F_ = 247.5 Hz).

*4-Methoxy-4′-methylbiphenyl* (**3dc**, Table 2, Entry 16). CAS: 53040-92-9; white solid (m.p. = 113–114 °C, lit. [59] 113–114 °C). ^1^H-NMR (CDCl_3_): δ 2.42 (s, 3H), 3.87 (s, 3H), 7.01 (d, *J* = 9.0 Hz, 2H), 7.27 (d, *J* = 9.0 Hz, 2H), 7.50 (d, *J* = 9.0 Hz, 2H), 7.56 (d, *J* = 9.0 Hz, 2H); ^13^C-NMR (CDCl_3_): δ 21.0, 55.2, 114.1 (2C), 126.5 (2C), 127.9 (2C), 129.4 (2C), 133.6, 136.3, 137.9, 158.9.

*4-Fluoro-4′-methoxybiphenyl* (**3eb**, Table 2, Entry 19 and Table 3, Entry 14). CAS: 450-39-5; white solid (m.p. = 88–90 °C, lit. [59] 88–90 °C). ^1^H-NMR (CDCl_3_): δ 3.84 (s, 3H), 6.94–6.99 (m, 2H), 7.07–7.13 (m, 2H), 7.44–7.51 (m, 4H); ^13^C-NMR (CDCl_3_): δ 55.3, 114.2 (2C), 115.5 (d, *J*_C-F_ = 21.8 Hz, 2C), 128.0 (2C), 128.2 (d, *J*_C-F_ = 7.5 Hz, 2C), 132.8, 136.9 (d, *J*_C-F_ = 3.0 Hz), 159.1, 162.0 (d, *J*_C-F_ = 243.8 Hz).

*4,4′-Dimethoxybiphenyl* (**3ec**, Table 2, Entry 20 and Table 3, Entry 15). CAS: 2132-80-1; pale yellow solid (m.p. = 178–180 °C, lit. [68] 178–180 °C). ^1^H-NMR (CDCl_3_): δ 3.84 (s, 6H), 6.93–6.97 (m, 4H), 7.45–7.49 (m, 4H); ^13^C-NMR (CDCl_3_): δ 55.3 (2C), 114.1 (4C), 127.7 (4C), 133.4 (2C), 158.6 (2C).

*4-Phenylphenol* (**3fa**, Table 3, Entries 16–18). CAS: 92-69-3; white solid (m.p. = 148–150 °C, lit. [59] 148–150 °C). ^1^H-NMR (DMSO–d_6_): δ 6.86 (d, *J* = 9.0 Hz, 2H), 7.25–7.28 (m, 1H), 7.36–7.57 (m, 6H), 9.57 (br, 1H); ^13^C-NMR (DMSO–d_6_): δ 115.7 (2C), 126.0 (2C), 126.4, 127.8 (2C), 128.8 (2C), 130.9, 140.2, 157.2.

*4-Hydroxy-4-fluorobiphenyl* (**3fb**, Table 3, Entry 19). CAS: 324-94-7; white solid (m.p. = 168–170 °C, lit. [71] 167–170 °C). ^1^H-NMR (DMSO–d_6_): δ 6.84 (d, *J* = 9.0 Hz, 2H), 7.21 (t, *J* = 9.0 Hz, 2H), 7.44 (d, *J* = 9.0 Hz, 2H), 7.55–7.60 (m, 2H), 9.56 (br, 1H); ^13^C-NMR (DMSO–d_6_): δ 115.5 (d, *J*_C-F_ = 21.0 Hz, 2C), 115.7 (2C), 127.7 (2C), 127.8 (d, *J*_C-F_ = 9.0 Hz, 2C), 130.0, 136.7 (d, *J*_C-F_ = 3.0 Hz), 157.1, 161.2 (d, *J*_C-F_ = 240.8 Hz).

*4-Hydroxy-4-methoxybiphenyl* (**3fc**, Table 3, Entry 20). CAS: 16881-71-3; white solid (m.p. = 173–175 °C, lit. [72] 171–173 °C). ^1^H-NMR (DMSO–d_6_): δ 3.75 (s, 3H), 6.82 (d, *J* = 9.0 Hz, 2H), 6.95 (d, *J* =9.0 Hz, 2H), 7.40 (d, *J* = 9.0 Hz, 2H), 7.48 (d, *J* = 9.0 Hz, 2H), 9.46 (br, 1H); ^13^C-NMR (DMSO–d_6_): δ 55.1, 114.2 (2C), 115.7 (2C), 127.0 (2C), 127.2 (2C), 130.7, 132.8, 156.5, 158.1.

*4′-Methylacetophenone* (**6a** [73], Table 4, Entries 1–4 and 7). CAS: 122-00-9; colorless liquid. ^1^H-NMR (CDCl_3_): δ 2.38 (s, 3H), 2.55 (s, 3H), 7.23 (d, *J* = 9.0 Hz, 2H), 7.83 (d, *J* = 8.3 Hz, 2H); ^13^C-NMR (CDCl_3_): δ 21.6, 26.5, 128.4 (2C), 129.2 (2C), 134.6, 143.8, 197.8.

*p-Tolunitrile* (**6b** [74], Table 4, Entries 5 and 8). CAS: 104-85-8; colorless liquid. ^1^H-NMR (CDCl_3_): δ 2.39 (s, 3H), 7.24 (d, *J* = 9.0 Hz, 2H), 7.51 (d, *J* = 9.0 Hz, 2H); ^13^C-NMR (CDCl_3_): δ 21.8, 109.2, 119.1, 129.8 (2C), 131.9 (2C), 143.6.

*4-Methylanisole* (**6e** [26], Table 4, Entry 6). CAS: 104-93-8; colorless liquid. ^1^H-NMR (CDCl_3_): δ 2.30 (s, 3H), 3.79 (s, 3H), 6.82 (d, *J* = 9.0 Hz, 2H), 7.10 (d, *J* = 9.0 Hz, 2H); ^13^C-NMR (CDCl_3_): δ 20.4, 55.2, 113.6 (2C), 129.7, 129.8 (2C), 157.4.

### 3.3. Procedure for Reuse of the Catalytic Aqueous Solution

The reaction was conducted following the procedure described in Section 3.2., and under the reaction conditions shown in Table 1, Entry 6. After the initial reaction, the aqueous reaction mixture was extracted with hexane (3 mL × 3) under vigorous stirring. The organic layer was separated from the aqueous phase by syringe, and the organic product was isolated from the combined organic phase according to a previously-described in Section 3.2. The residual aqueous solution was then charged with **1a**, **2a**, and NaHCO_3_ for the next reaction.

## 4. Conclusions

In conclusion, we have proved that the PdCl_2_(NH_3_)_2_/cationic 2,2′-bipyridyl system is highly efficient and provides a reusable catalyst for Stille couplings. This catalytic system exhibits a high efficiency for the coupling of aryl iodides and activated aryl bromides with various aryltributylstannanes under a very low catalyst loading (1 ppm). This water-compatible catalytic system enables the reaction to be conducted by a very simple procedure, allowing easy separation of the catalytic aqueous solution from the organic products, rendering it very suitable for practical applications.

## Figures and Tables

**Table 1:** Optimization studies for the Pd(NH_3_)_2_Cl_2_/**L**-catalyzed Stille coupling of 4′-iodoacetophenone (**1a**) and PhSnBu_3_ (**2a**) in water ^a^.

Entry	Base	Yield (%) ^b^
1	KOH	70
2	K_3_PO_4_	75
3	K_2_CO_3_	85
4	KF	60
5	KOAc	74
6	NaHCO_3_	95
7 ^c^	NaHCO_3_	32
8 ^d^	NaHCO_3_	35
9 ^e^	NaHCO_3_	0
10 ^f^	NaHCO_3_	90
11 ^g^	NaHCO_3_	95

^a^ Reaction conditions: **1a** (1 mmol), **2a** (1.2 mmol), base (2 mmol), PdCl_2_(NH_3_)_2_/L (1 mol %), H_2_O (3 mL) at 110 °C for 0.5 h; ^b^ Isolated yields; ^c^ In the absence of **L**; ^d^ 2,2′-Bipyridyl was used as the ligand; ^e^ 2,2′-Bipyridine-4,4′-dicarboxylic acid was used as the ligand; ^f^ PhSnCl_3_ (1.2 mmol) was used; ^g^ PhSnMe_3_ (1.2 mmol) was used.

**Table 2:** Pd-catalyzed Stille coupling of aryl iodides (**1**) and aryltributylstannane (**2**) in water ^a^.

Entry	Aryl Iodide	ArSnBu_3_	[Pd] (mol %)	Duration (h)	Yield (%) ^b^
1	4-IC_6_H_4_COMe **1a**	C_6_H_5_SnBu_3_ **2a**	0.01	3	**3aa** (98)
2 ^c^	**1a**	**2a**	0.0001	48	**3aa** (82)
3	**1a**	4-FC_6_H_5_SnBu_3_ **2b**	0.01	3	**3ab** (97)
4	**1a**	4-MeOC_6_H_5_SnBu_3_ **2c**	0.01	3	**3ac** (93)
5 ^c^	**1a**	**2c**	0.0001	48	**3ac** (72)
6	4-IC_6_H_4_CN **1b**	**2a**	0.01	3	**3ba** (91)
7 ^c^	**1b**	**2a**	0.0001	48	**3ba** (75)
8	**1b**	**2b**	0.01	3	**3bb** (93)
9	**1b**	**2c**	0.01	3	**3bc** (90)
10	C_6_H_5_I **1c**	**2a**	0.01	6	**3ca** (95)
11	**1c**	**2b**	0.01	6	**3cb** (95)
12	**1c**	**2c**	0.01	6	**3cc** (90)
13	4-IC_6_H_4_Me **1d**	**2a**	0.01	6	**3da** (89)
14 ^c^	**1d**	**2a**	0.0001	48	**3da** (59)
15	**1d**	**2b**	0.01	6	**3db** (82)
16	**1d**	**2c**	0.01	6	**3dc** (88)
17	4-IC_6_H_4_OMe **1e**	**2a**	0.01	6	**3cc** (94)
18 ^c^	**1e**	**2a**	0.0001	48	**3cc** (66)
19	**1e**	**2b**	0.01	6	**3eb** (84)
20	**1e**	**2c**	0.01	6	**3ec** (80)

^a^ *Reaction conditions*: **1** (1 mmol), **2** (1.2 mmol), NaHCO_3_ (2 mmol), H_2_O (3 mL) at 110 °C; ^b^ Isolated yields; ^c^ **1** (10 mmol), **2** (12 mmol), NaHCO_3_ (20 mmol), H_2_O (30 mL) at 110 °C.

**Table 3:** Pd-catalyzed Stille coupling of aryl bromides (**4**) and aryltributylstannane (**2**) in water ^a^.

Entry	Aryl Bromide	ArSnBu_3_	[Pd] (mol %)	Duration (h)	Yield (%) ^b^
1	4-BrC_6_H_4_COMe **4a**	C_6_H_5_SnBu_3_ **2a**	0.01	6	**3aa** (96)
2 ^c^	**4a**	**2a**	0.0001	48	**3aa** (70)
3	**4a**	4-FC_6_H_5_SnBu_3_ **2b**	0.01	6	**3ab** (96)
4	**4a**	**2b**	0.001	12	**3ab** (91)
5	**4a**	4-MeOC_6_H_5_SnBu_3_ **2c**	0.01	6	**3ac** (92)
6	**4a**	**2c**	0.001	12	**3ac** (95)
7	4-BrC_6_H_4_CN **4b**	**2a**	0.01	6	**3ba** (93)
8	**4b**	**2b**	0.01	8	**3bb** (90)
9	**4b**	**2c**	0.01	8	**3bc** (88)
10	C_6_H_5_Br **4c**	**2a**	1	24	**3ca** (92)
11	**4c**	**2b**	1	36	**3cb** (96)
12	**4c**	**2c**	1	36	**3cc** (94)
13	4-BrC_6_H_4_OMe **4e**	**2a**	1	24	**3cc** (84)
14	**4e**	**2b**	1	24	**3eb** (83)
15	**4e**	**2c**	1	36	**3ec** (97)
16 ^d^	4-BrC_6_H_4_OH **4f**	**2a**	1	3	**3fa** (90)
17 ^d^	**4f**	**2a**	0.01	12	**3fa** (96)
18 ^c,d^	**4f**	**2a**	0.0001	48	**3fa** (56)
19 ^d^	**4f**	**2b**	0.01	12	**3fb** (94)
20 ^d^	**4f**	**2c**	0.01	12	**3fc** (88)

^a^ Reaction conditions: **4** (1 mmol), **2** (1.2 mmol), NaHCO_3_ (2 mmol), H_2_O (3 mL) at 110 °C; ^b^ Isolated yields; ^c^ **4** (10 mmol), **2** (12 mmol), NaHCO_3_ (20 mmol), H_2_O (30 mL) at 110 °C; ^d^ 3 equiv. of NaHCO_3_ was applied.

**Table 4:** Pd-catalyzed Stille coupling of aryl halides (**1** or **4**) and tetramethylstannane (**5**) in water ^a^.

Entry	Aryl Halide	Duration (h)	Yield (%) ^b^
1 ^c^	4-IC_6_H_4_COMe **1a**	24	**6a** (51)
2 ^d^	**1a**	24	**6a** (63)
3 ^e^	**1a**	24	**6a** (70)
4	**1a**	24	**6a** (91)
5	4-IC_6_H_4_CN **1b**	24	**6b** (78)
6	4-IC_6_H_4_OMe **1e**	36	**6e** (42)
7	4-BrC_6_H_4_COMe **4a**	48	**6a** (40)
8	4-BrC_6_H_4_CN **4b**	48	**6b** (31)

^a^ Reaction conditions: **1** or **4** (1 mmol), **5** (2 mmol), PdCl_2_(NH_3_)_2_/**L** (1 mol %), tetraethylammonium iodide (TEAI, 1 mmol), NaHCO_3_ (2 mmol), H_2_O (3 mL) at 110 °C; ^b^ Isolated yields; ^c^ In the absence of TEAI; ^d^ Tetrabutylammonium bromide (TBAB, 1 mmol) was used. ^e^ Tetrabutylammonium hydroxyl (TBAOH, 1 mmol) was used.

## Supplementary Materials

The following are available online at: http://www.mdpi.com/1420-3049/21/9/1025/s1: copies of ^1^H- and ^13^C-NMR spectra of all Stille coupling products.
