# Supplementary material for: A Highly Efficient and Reusable Palladium(II)/Cationic 2,2’-Bipyridyl-Catalyzed Stille Coupling in Water
Source: Molecules. 2016 Sep 9;21(9):1205. doi: 10.3390/molecules21091205 (PMC6273891; doi:10.3390/molecules21091205)
Supplement: Supplementary file 1 [file molecules-21-01205-s001.pdf]

Supplementary Materials: Highly Efficient and Reusable Palladium(II)/Cationic 2,2′-Bipyridyl-Catalyzed Stille Coupling in Water

Wei-Yi Wu, Ling-Jun Liu, Fen-Ping Chang, Yu-Lun Cheng and Fu-Yu Tsai

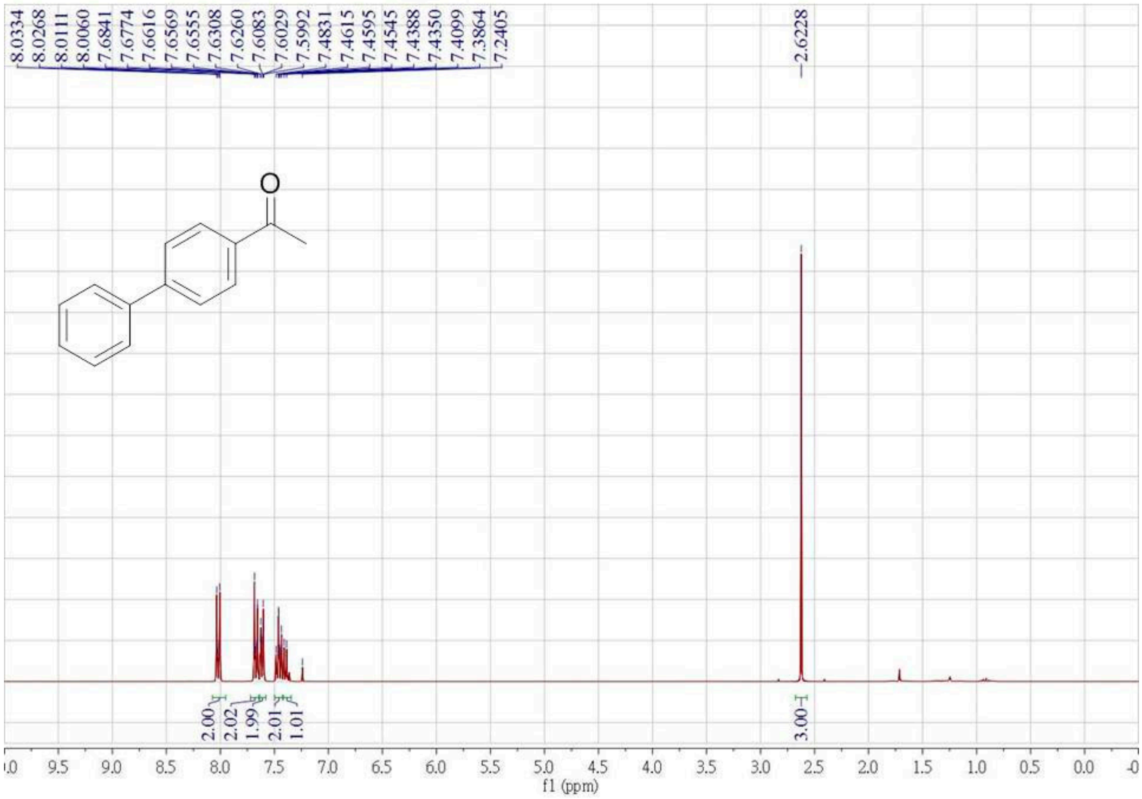

Figure S1. ^{1}H-NMR spectrum of compound **3aa**.

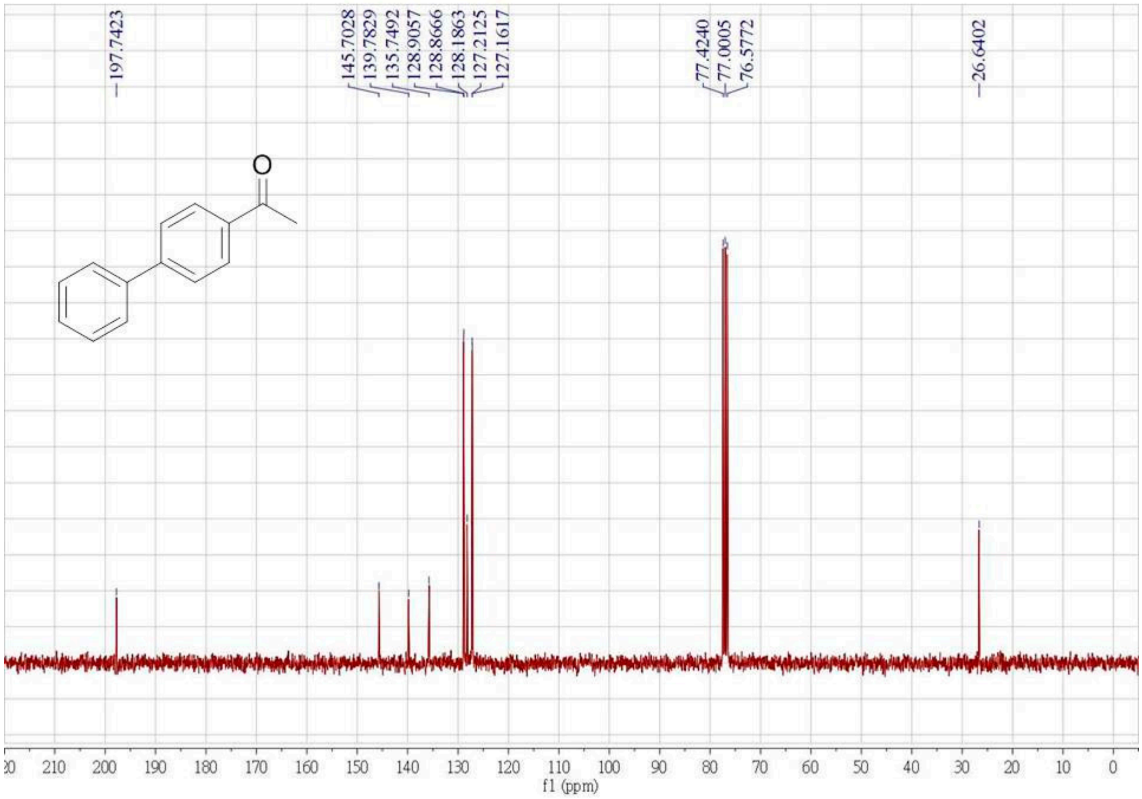

Figure S2. ^{13}C-NMR of compound **3aa**.

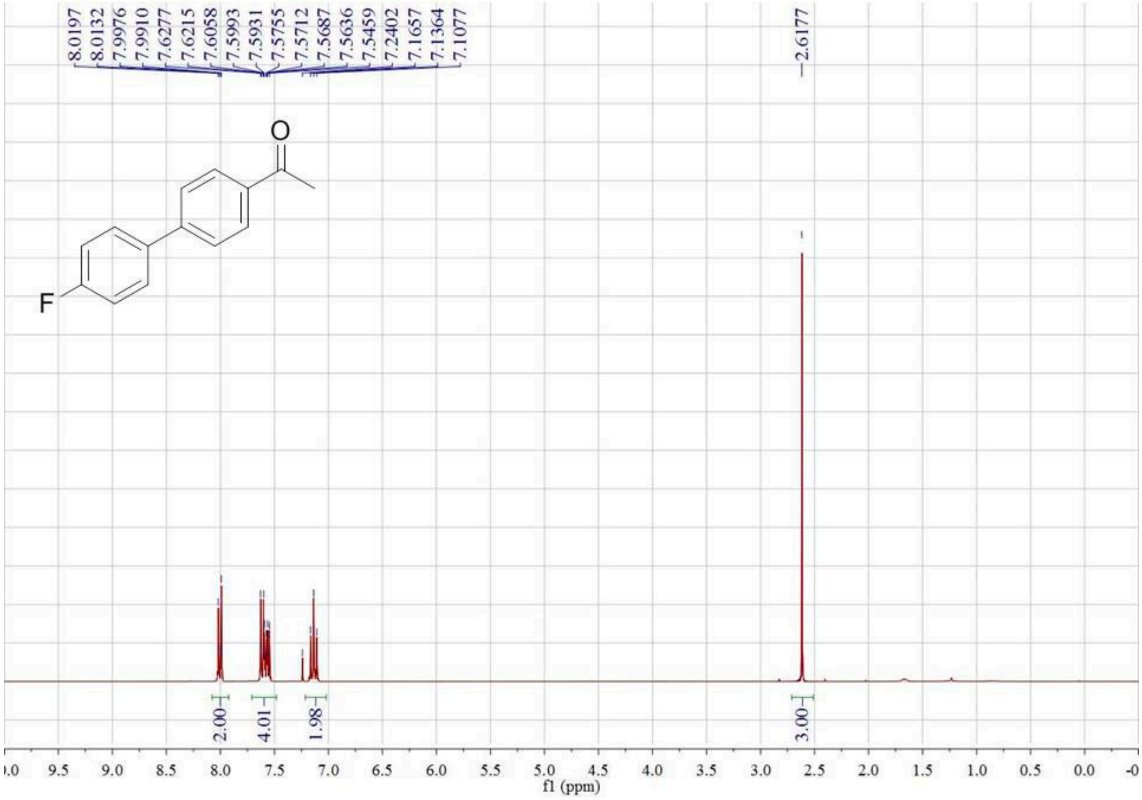

Figure S3. ^{1}H-NMR spectrum of compound **3ab**.

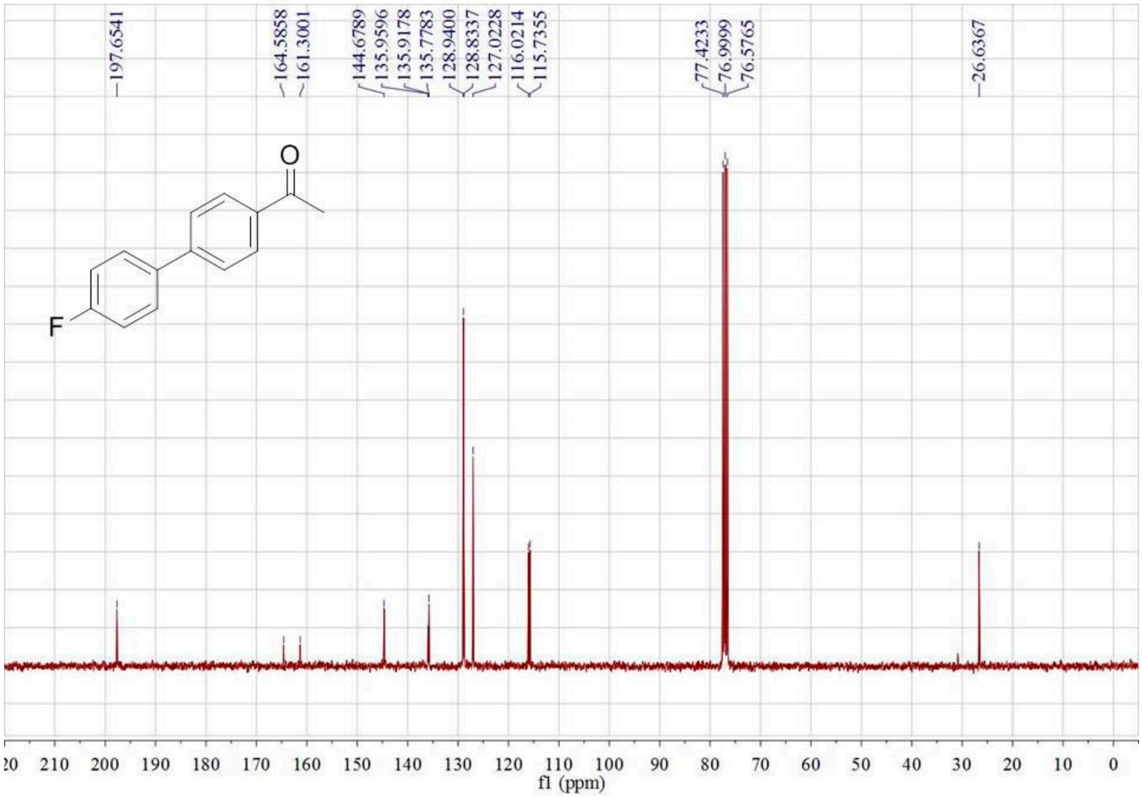

Figure S4. ^{13}C-NMR of compound **3ab**.

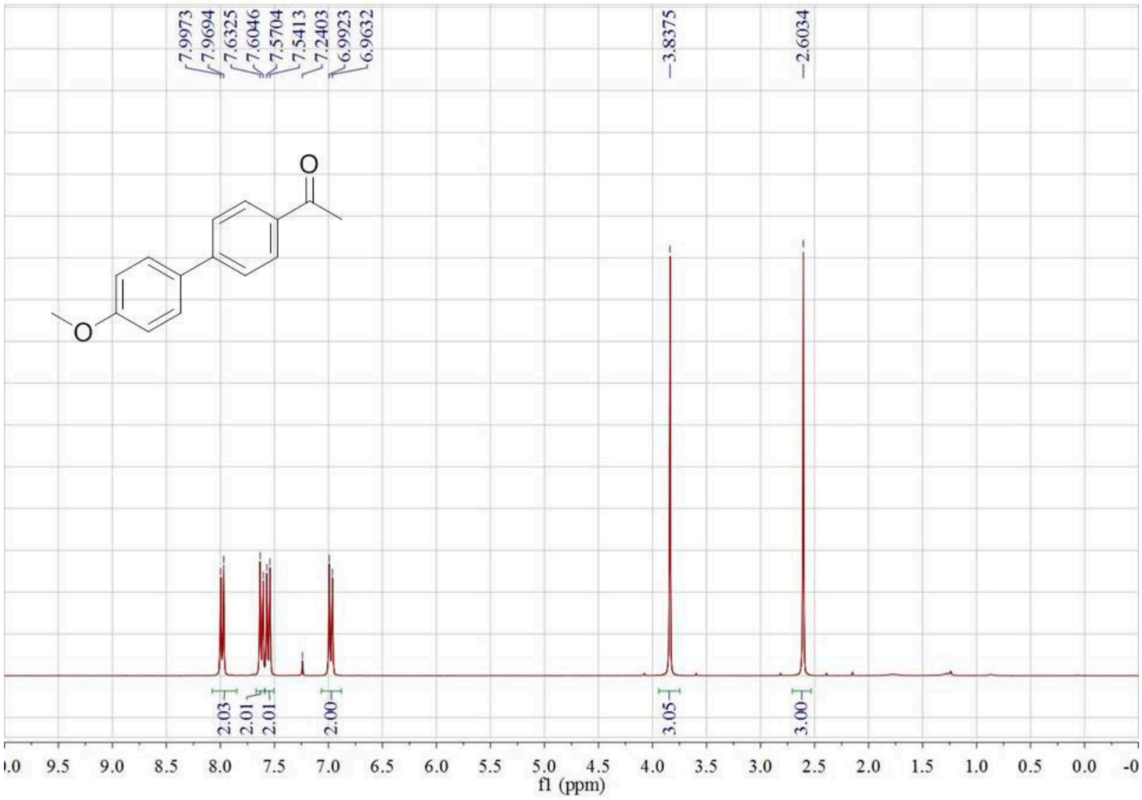

Figure S5. ^{1}H-NMR spectrum of compound **3ac**.

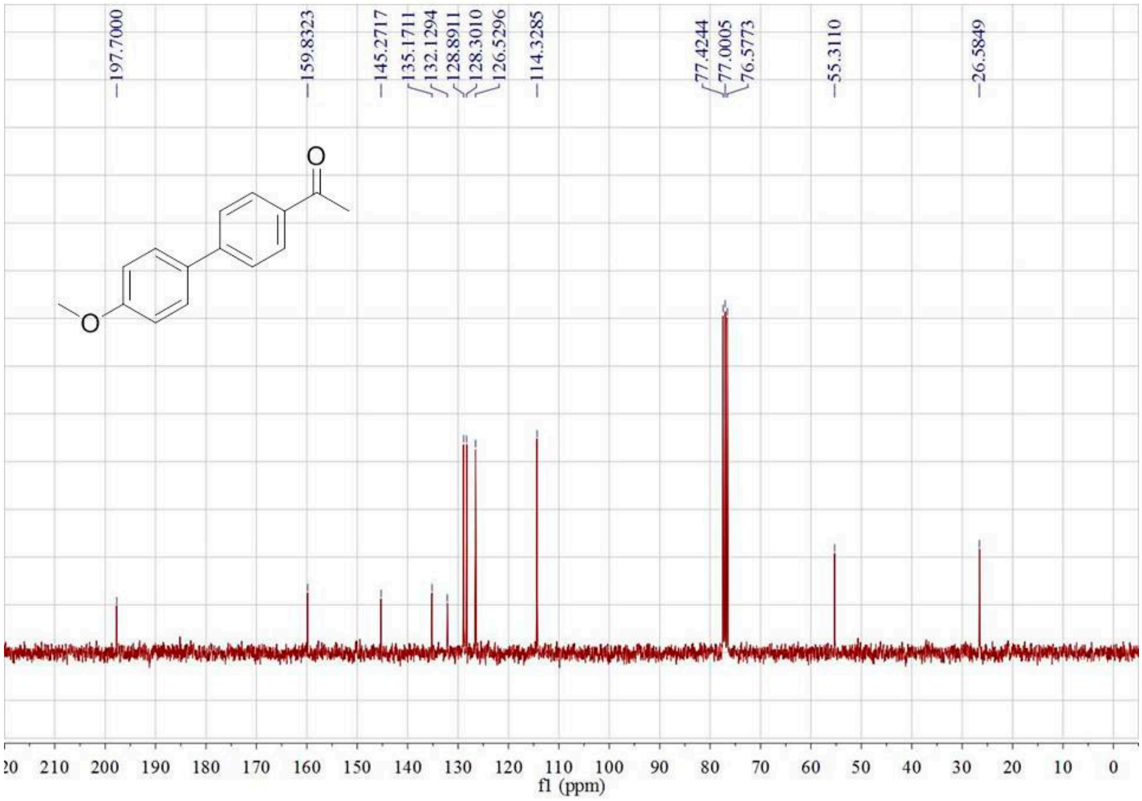

Figure S6. ^{13}C-NMR of compound **3ac**.

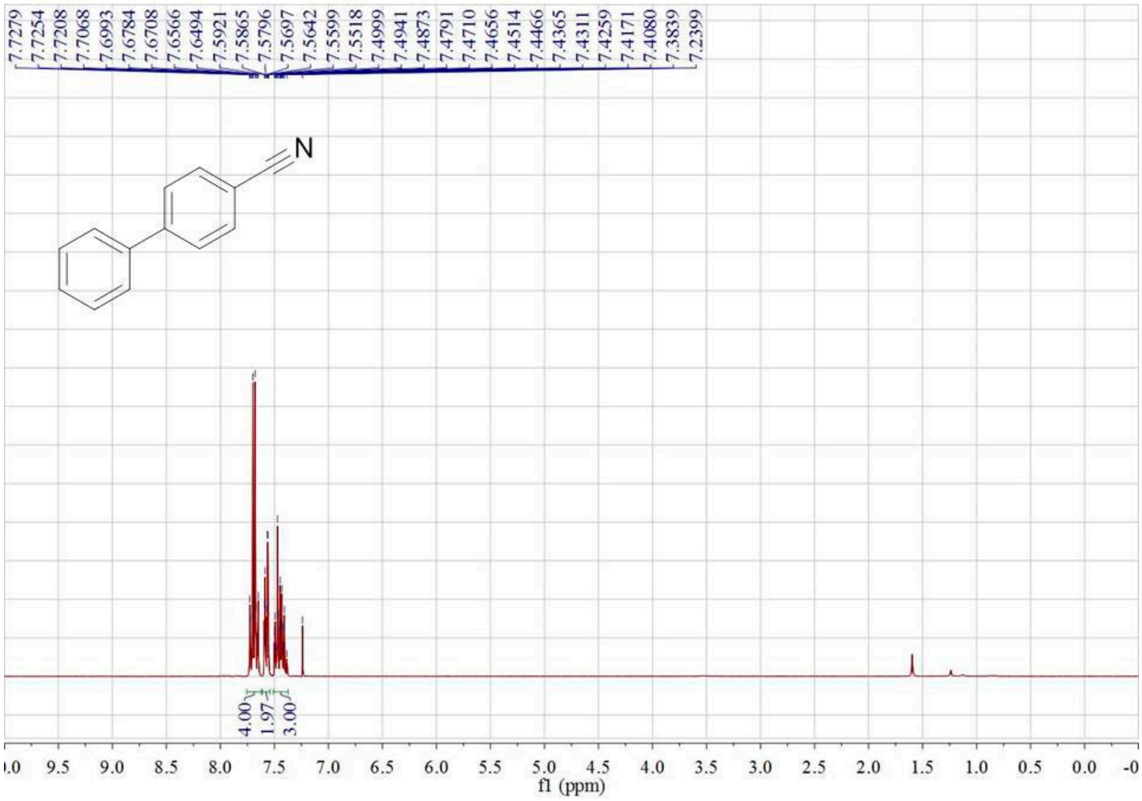

Figure S7. ^{1}H-NMR of compound **3ba**.

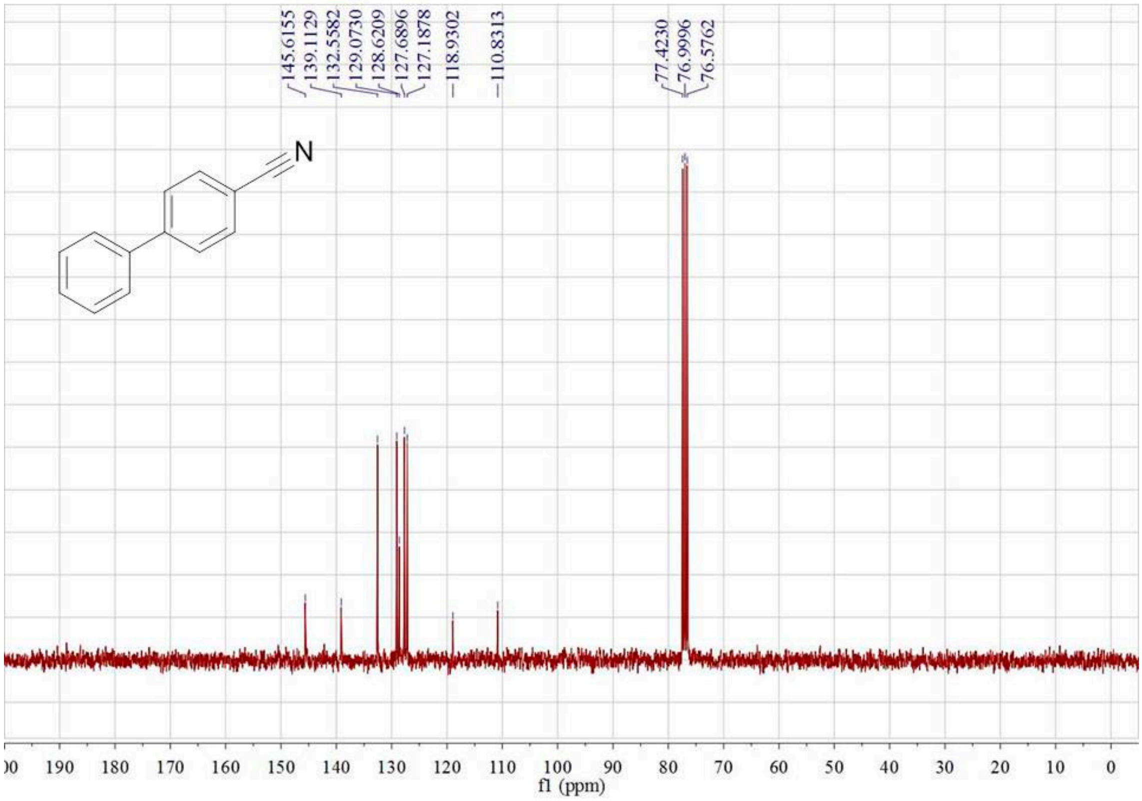

Figure S8. ^{13}C-NMR of compound **3ba**.

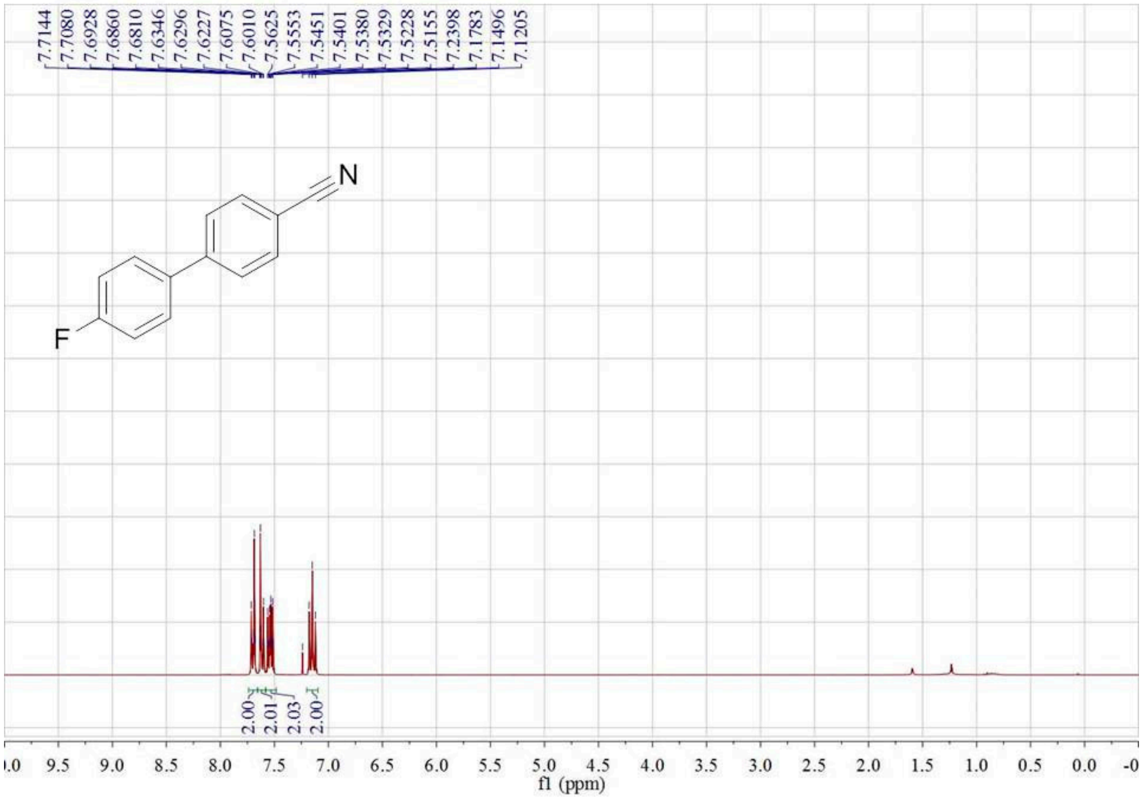

Figure S9. ^{1}H-NMR of compound **3bb**.

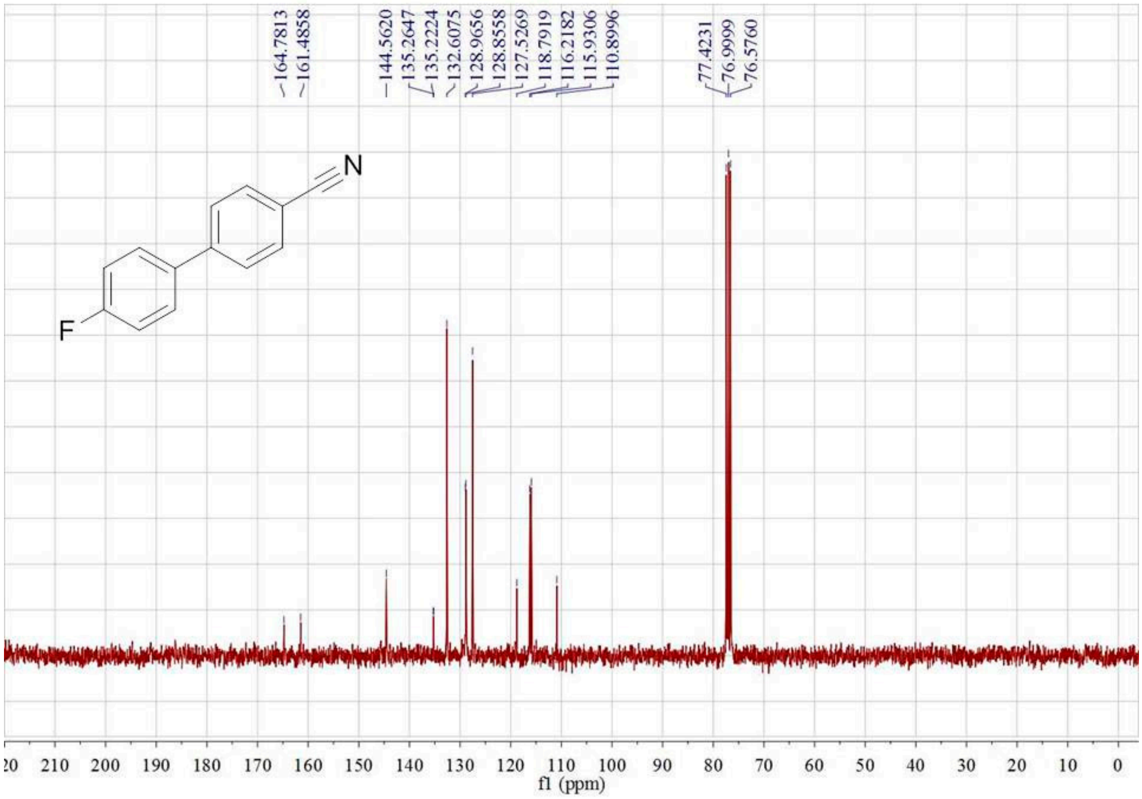

Figure S10. ^{13}C-NMR of compound **3bb**.

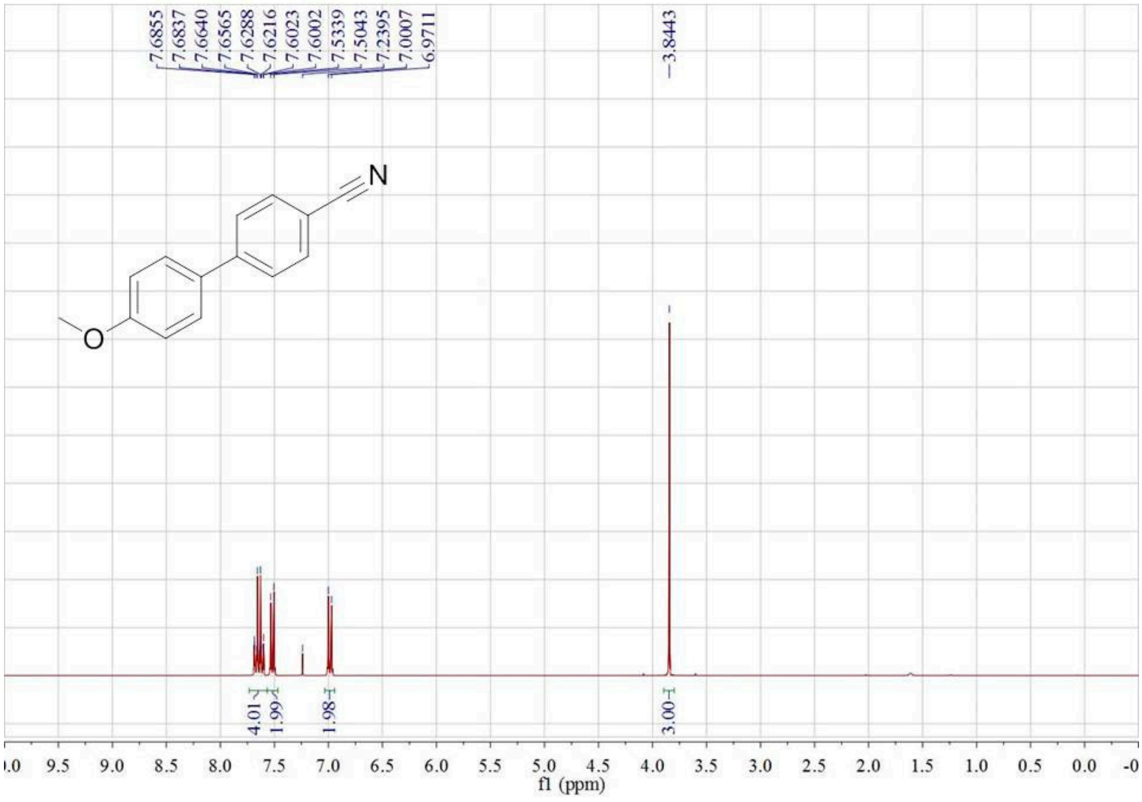

Figure S11. ^{1}H-NMR of compound **3bc**.

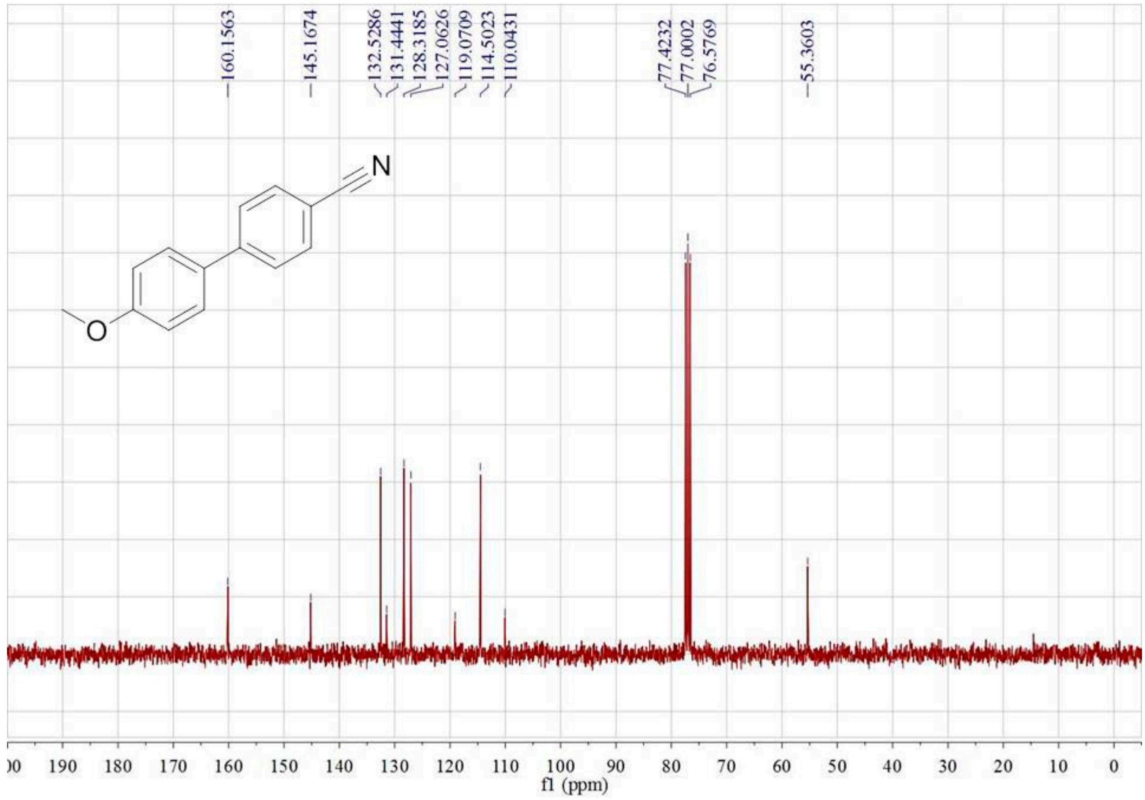

Figure S12. ^{13}C-NMR of compound **3bc**.

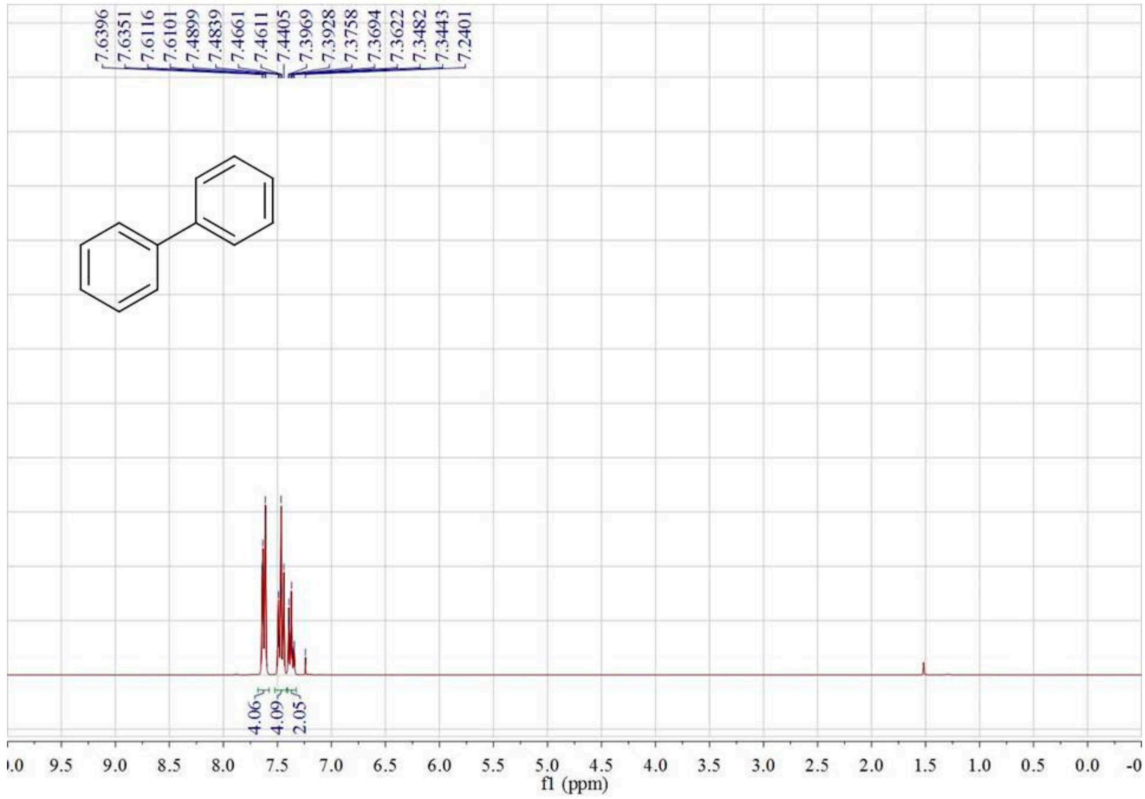

Figure S13. ^{1}H-NMR of compound **3ca**.

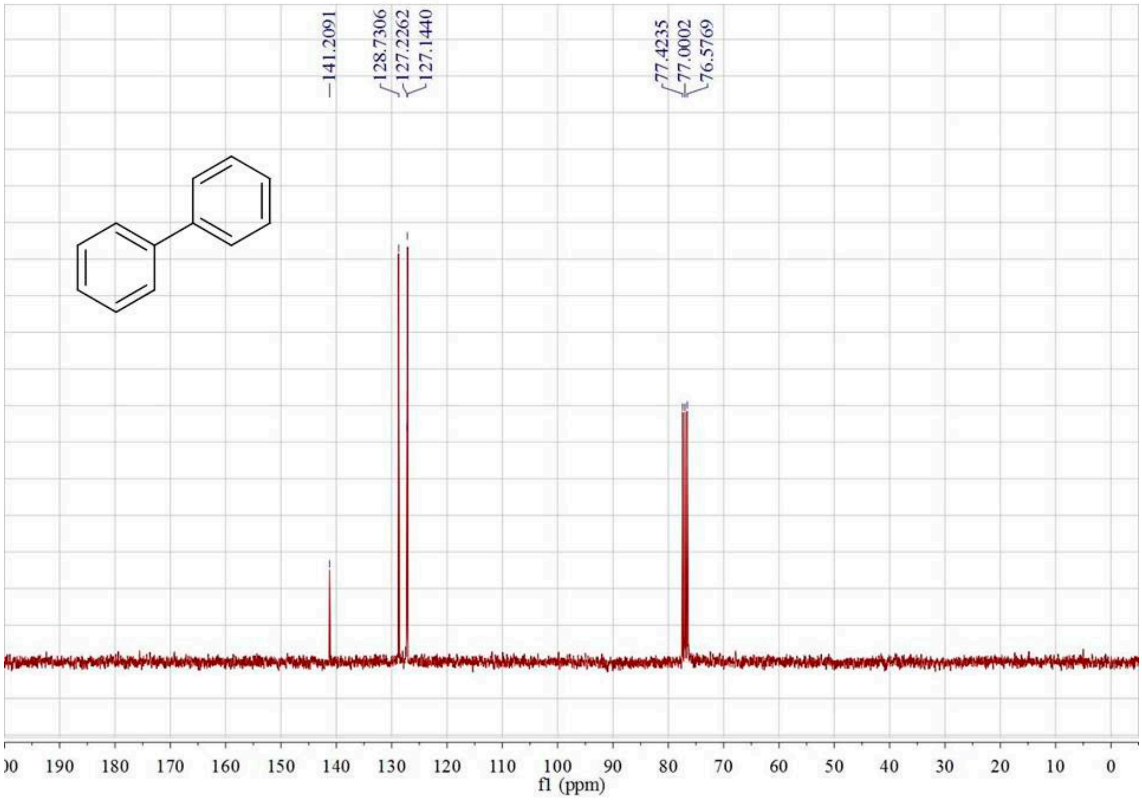

Figure S14. ^{13}C-NMR of compound **3ca**.

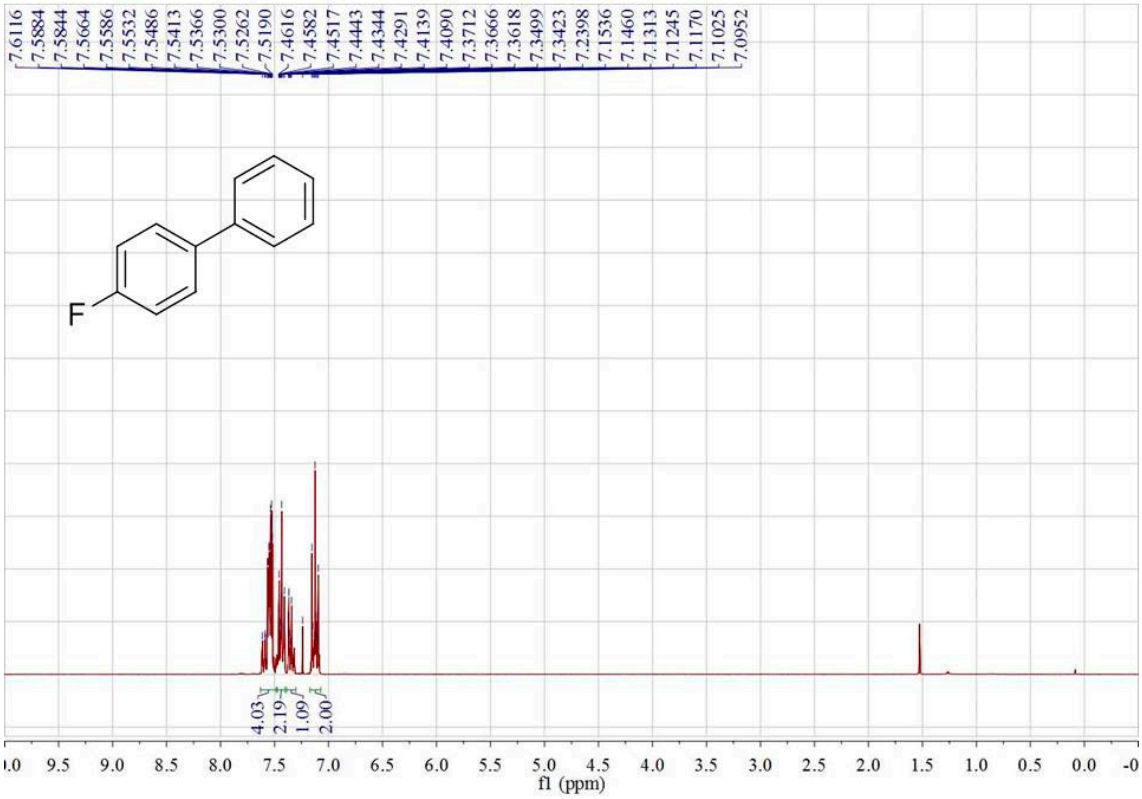

Figure S15. ^{1}H-NMR of compound **3cb**.

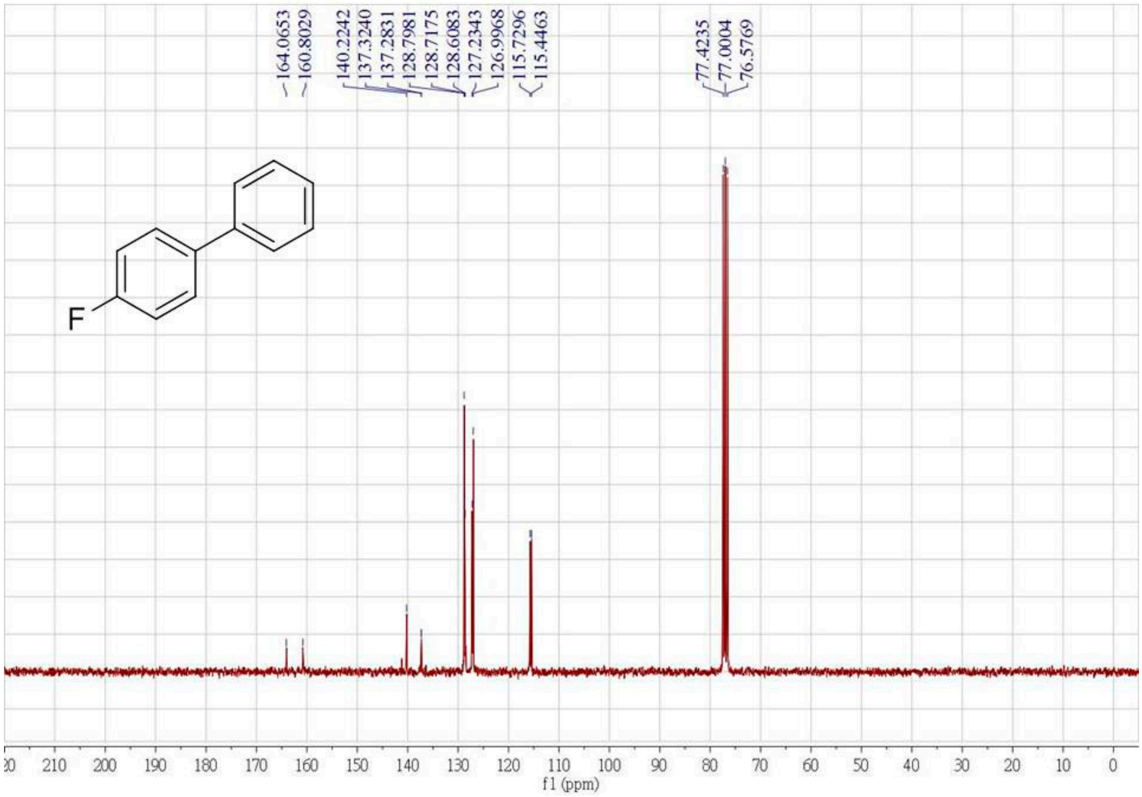

Figure S16. ^{13}C-NMR of compound **3cb**.

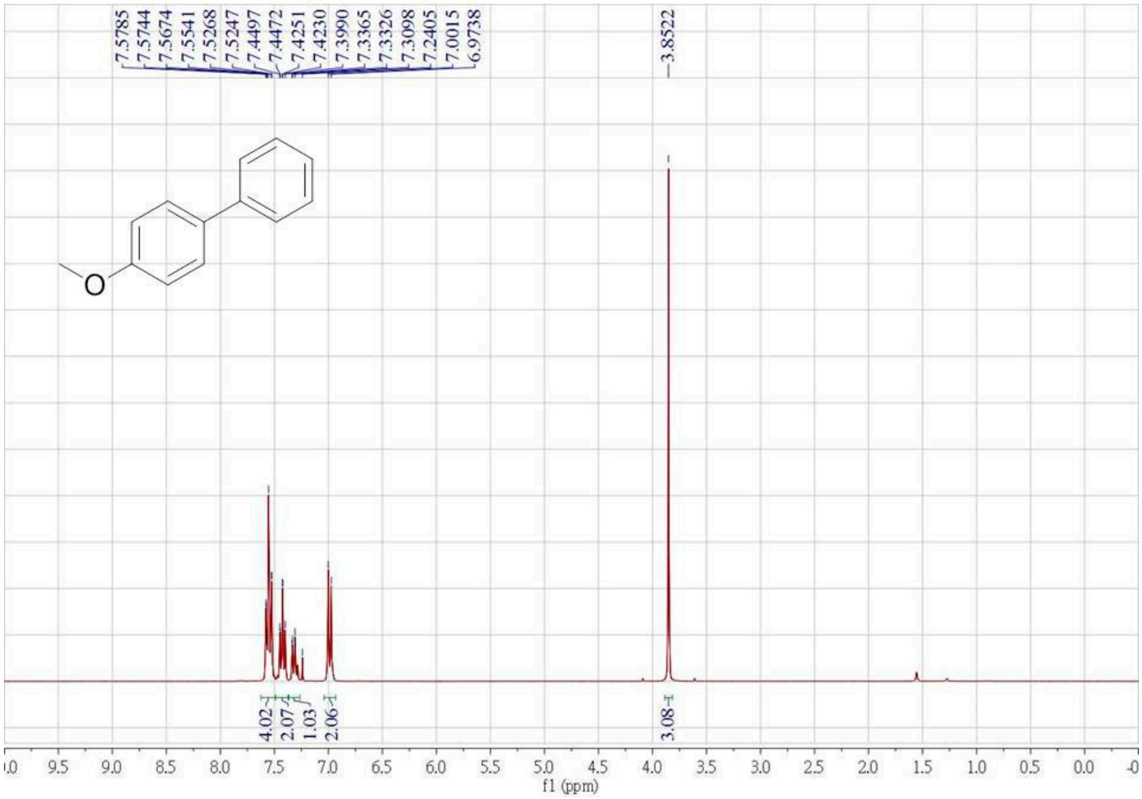

Figure S17. ^{1}H-NMR of compound **3cc**.

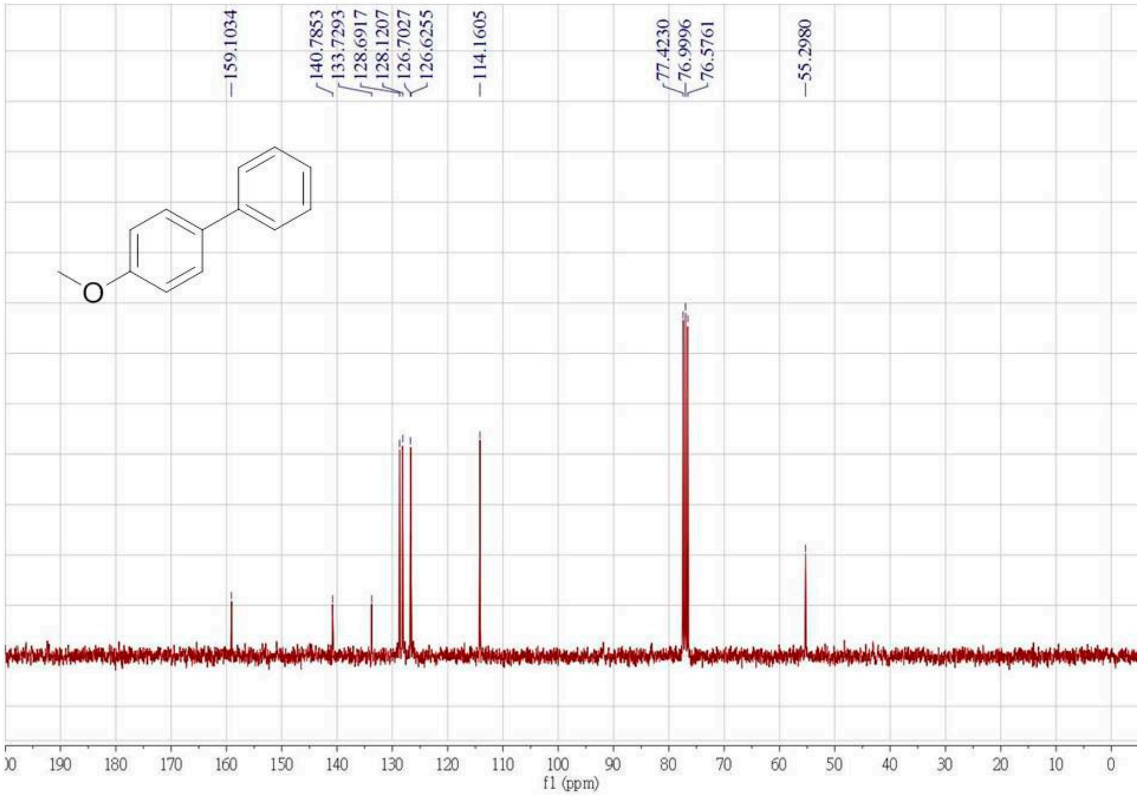

Figure S18. ^{13}C-NMR of compound **3cc**.

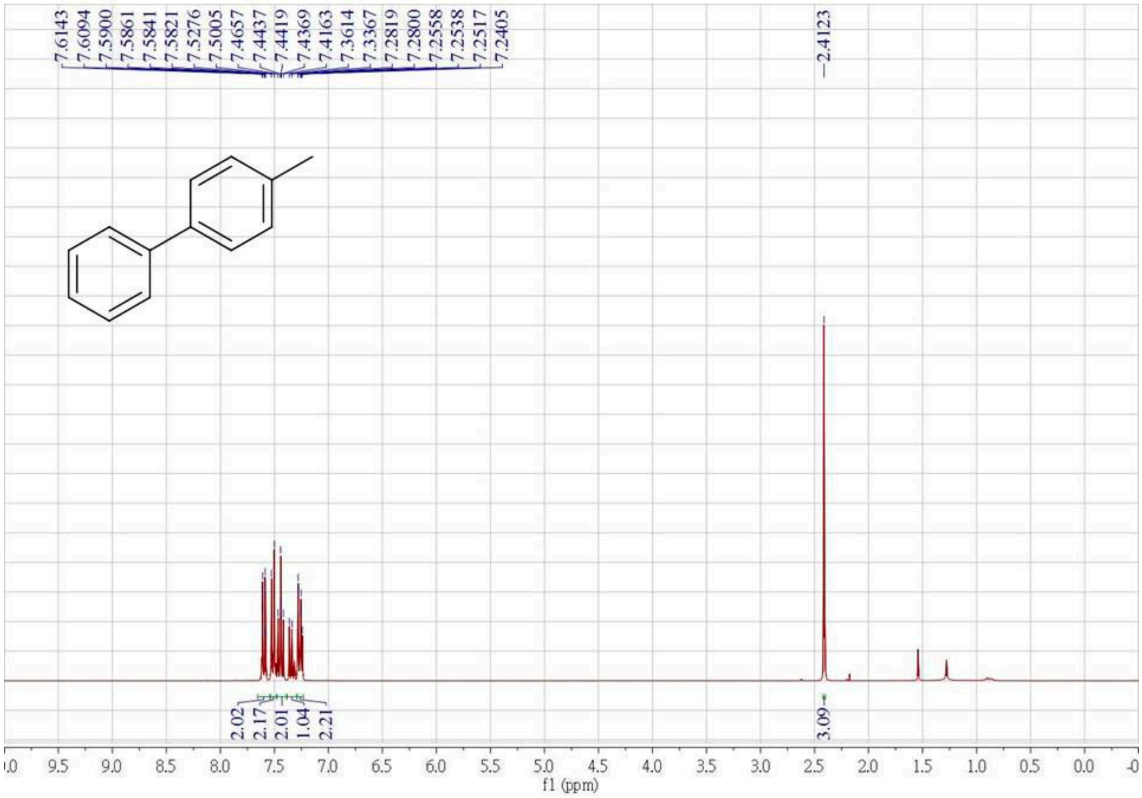

Figure S19. ^{1}H-NMR of compound **3da**.

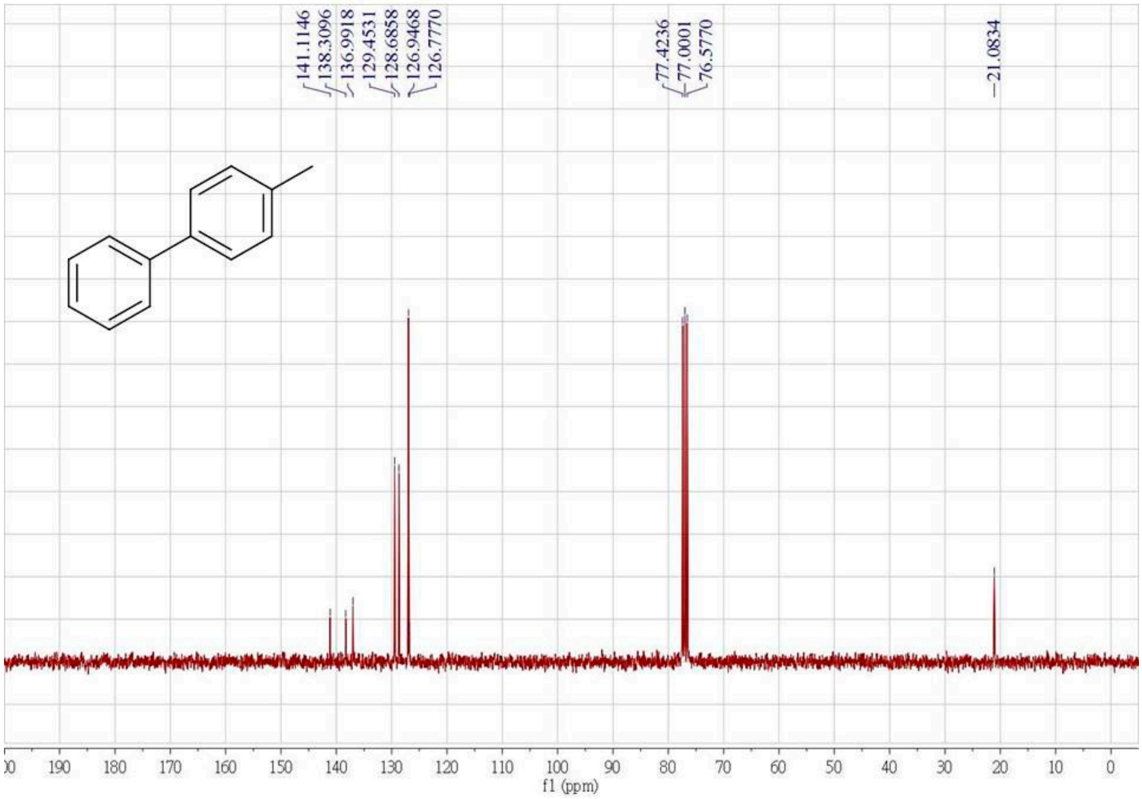

Figure S20. ^{13}C-NMR of compound **3da**.

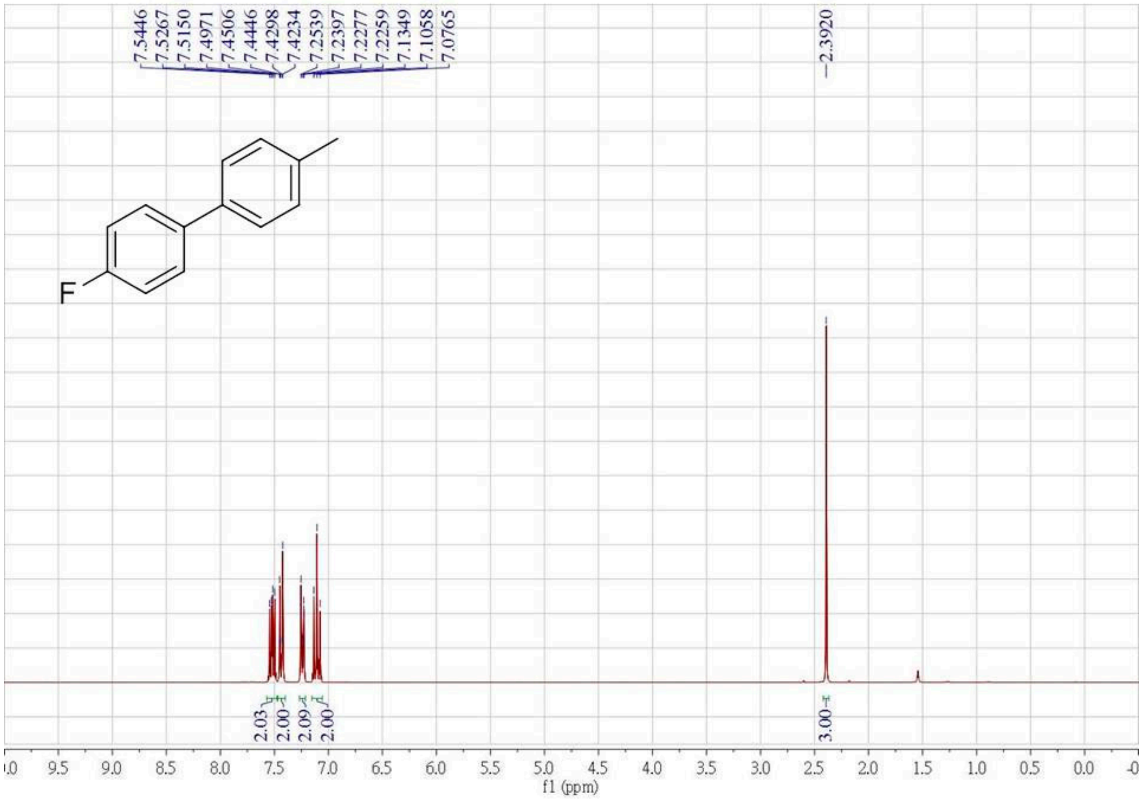

Figure S21. ^{1}H-NMR of compound **3db**.

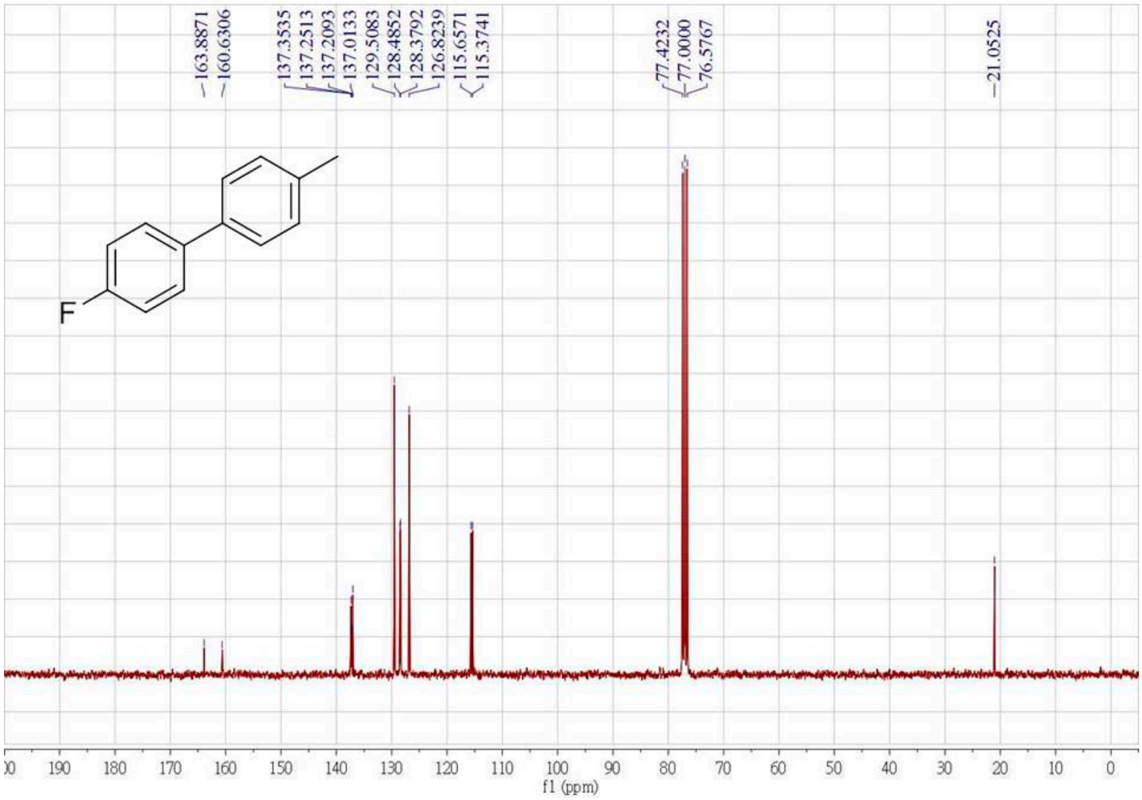

Figure S22. ^{13}C-NMR of compound **3db**.

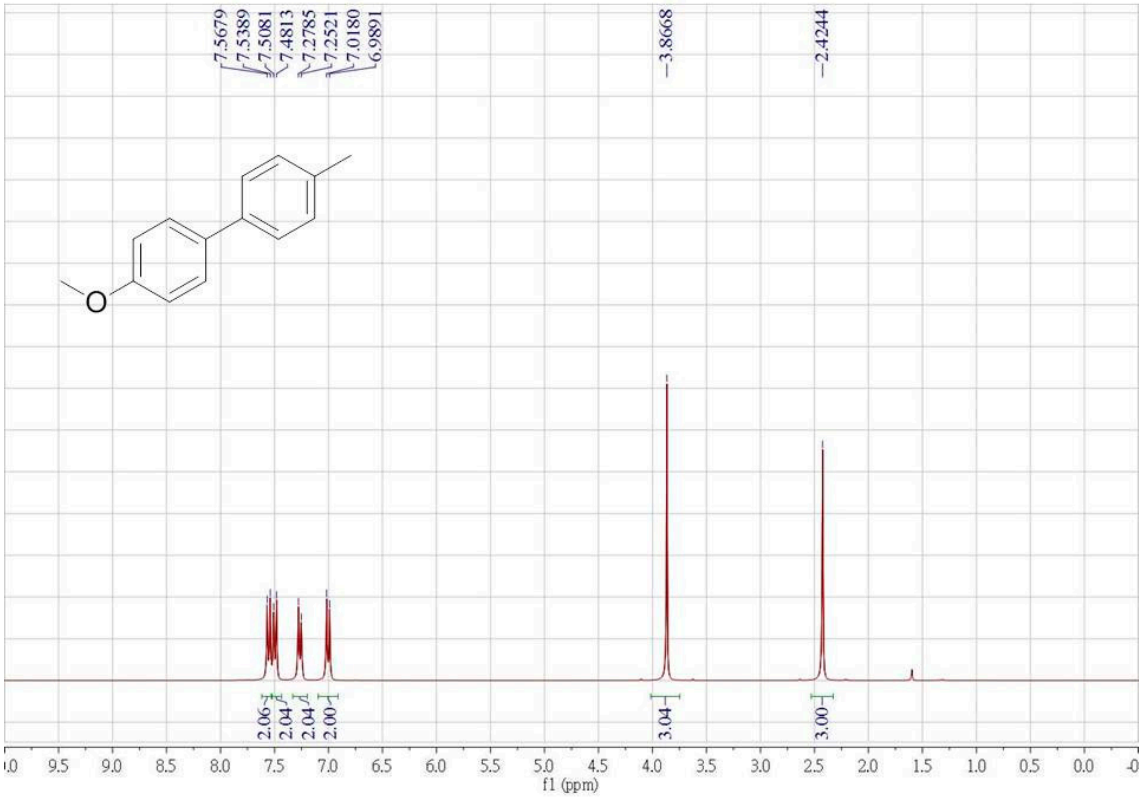

Figure S23. ^{1}H-NMR of compound **3dc**.

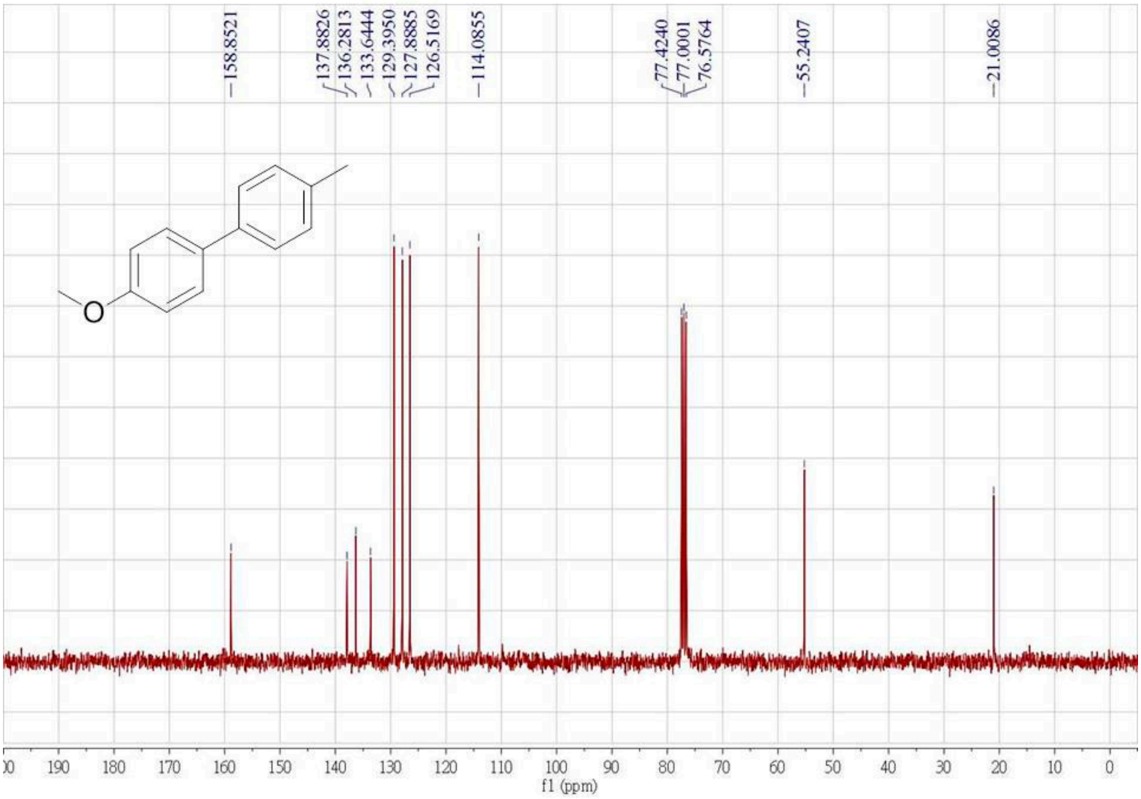

Figure S24. ^{13}C-NMR of compound **3dc**.

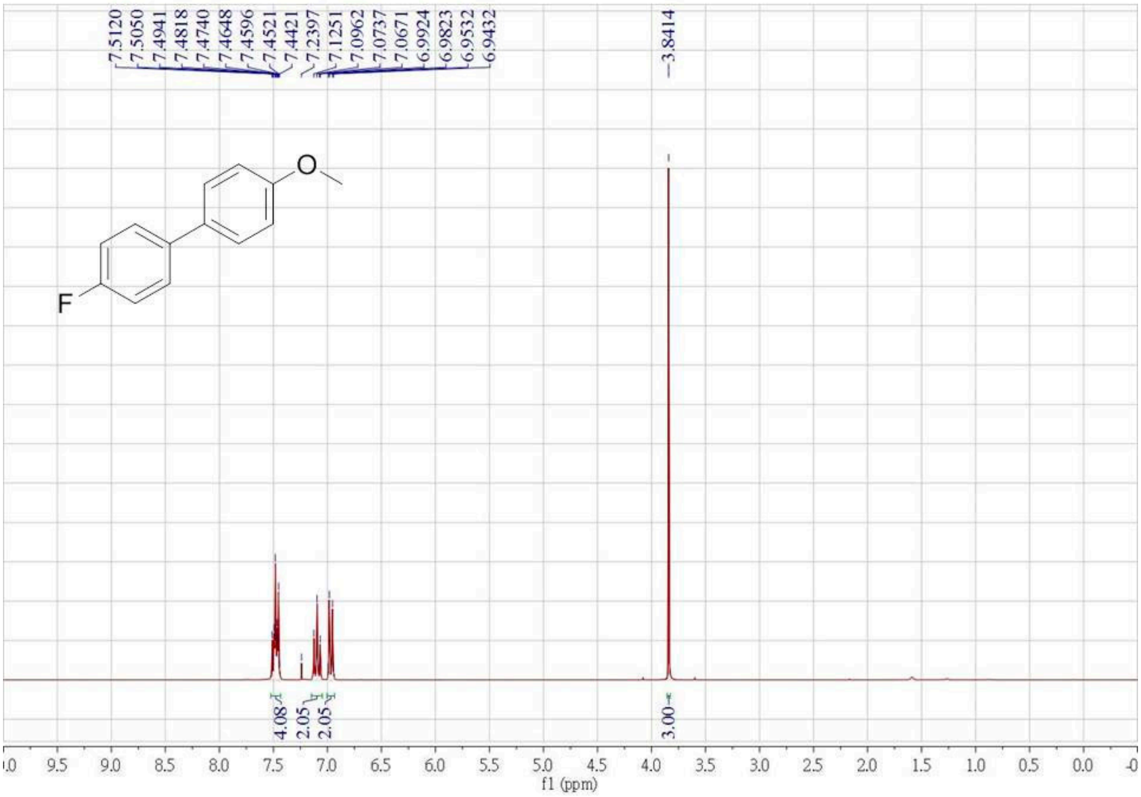

Figure S25. ^{1}H-NMR of compound **3eb**.

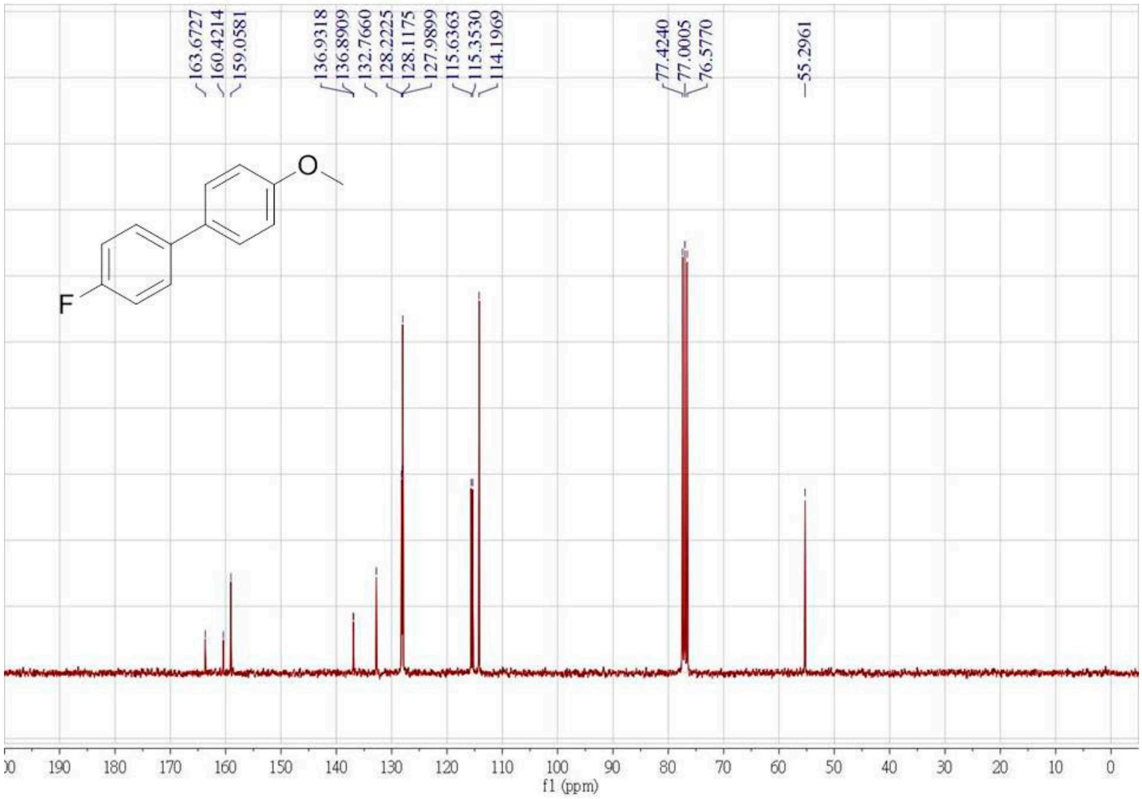

Figure S26. ^{13}C-NMR of compound **3eb**.

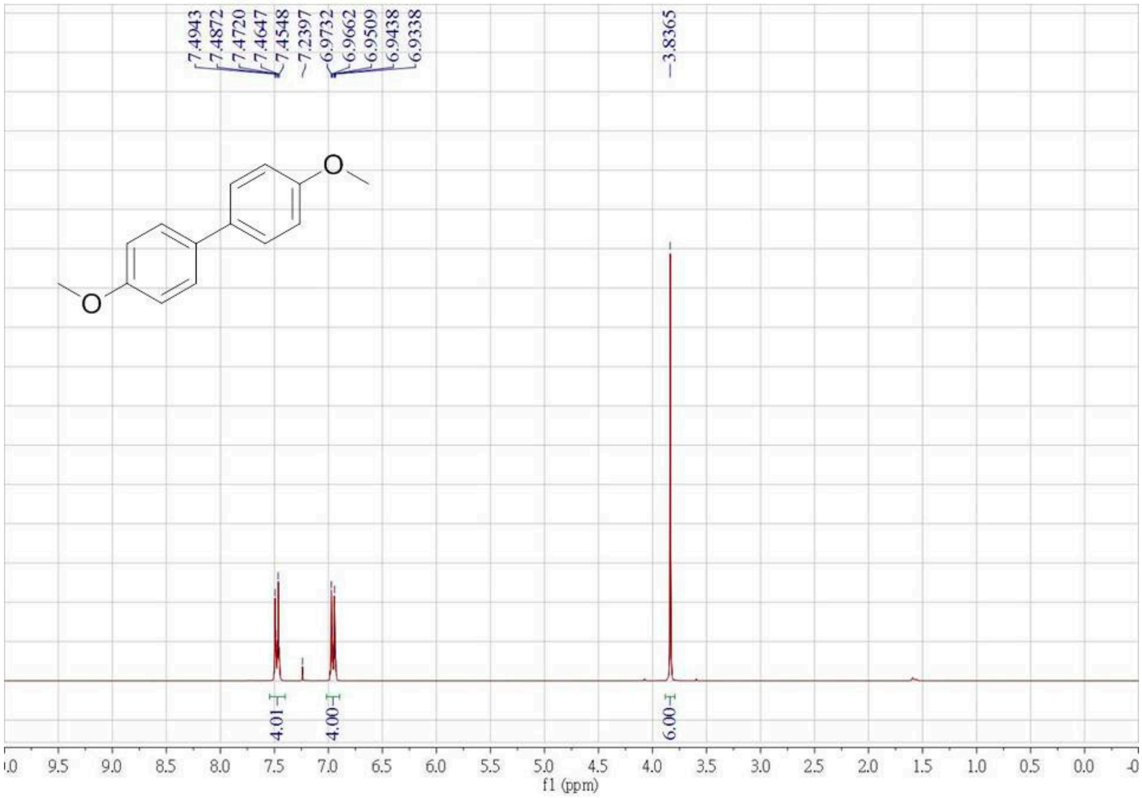

Figure S27. ^{1}H-NMR of compound **3ec**.

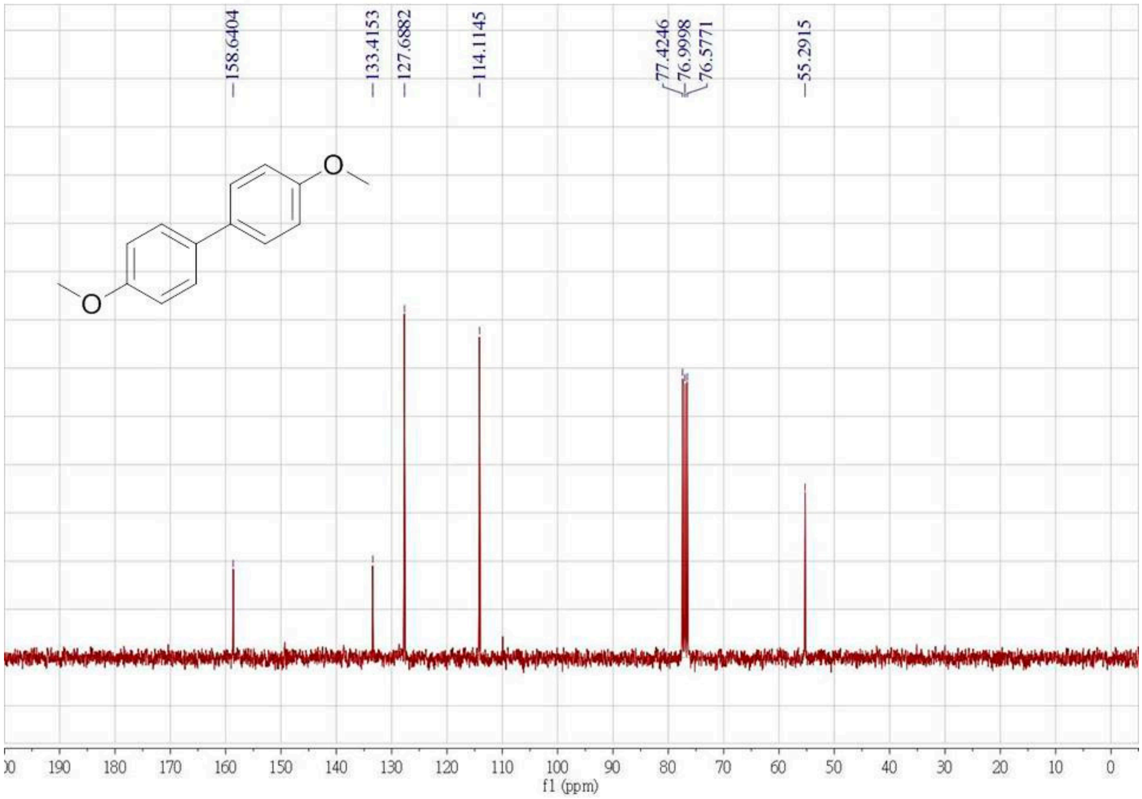

Figure S28. ^{13}C-NMR of compound **3ec**.

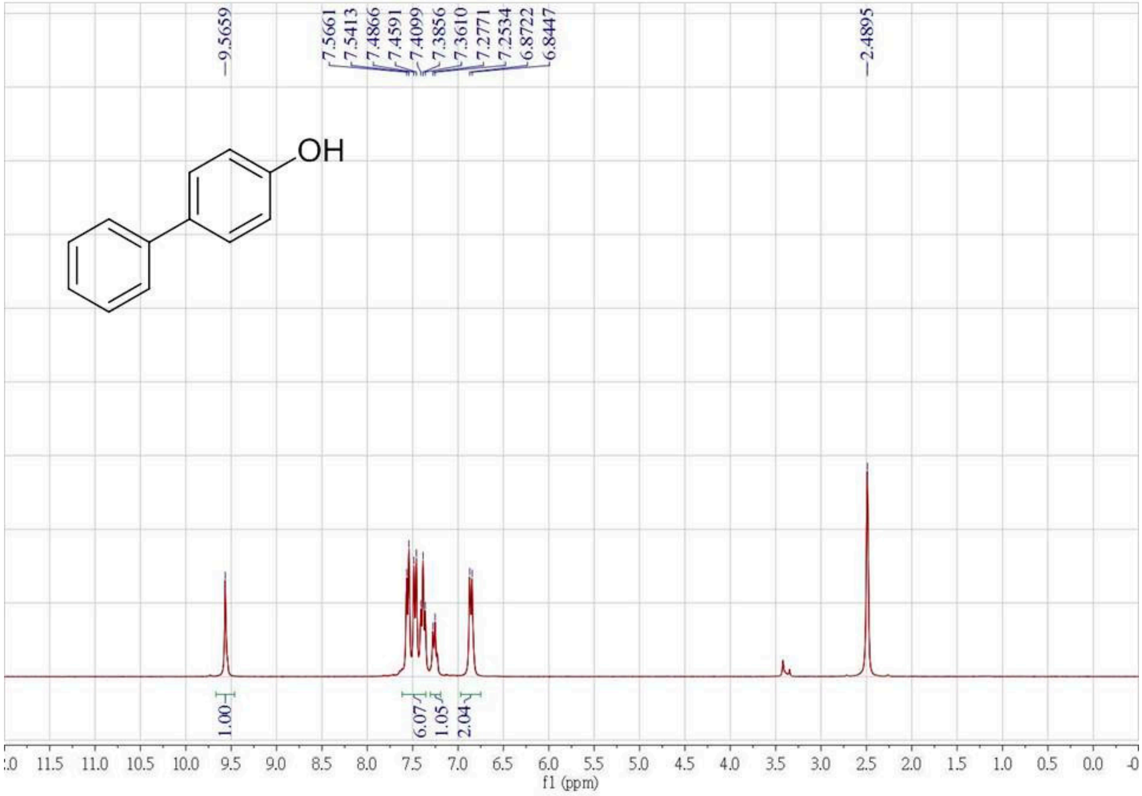

Figure S29. ^{1}H-NMR of compound **3fa**.

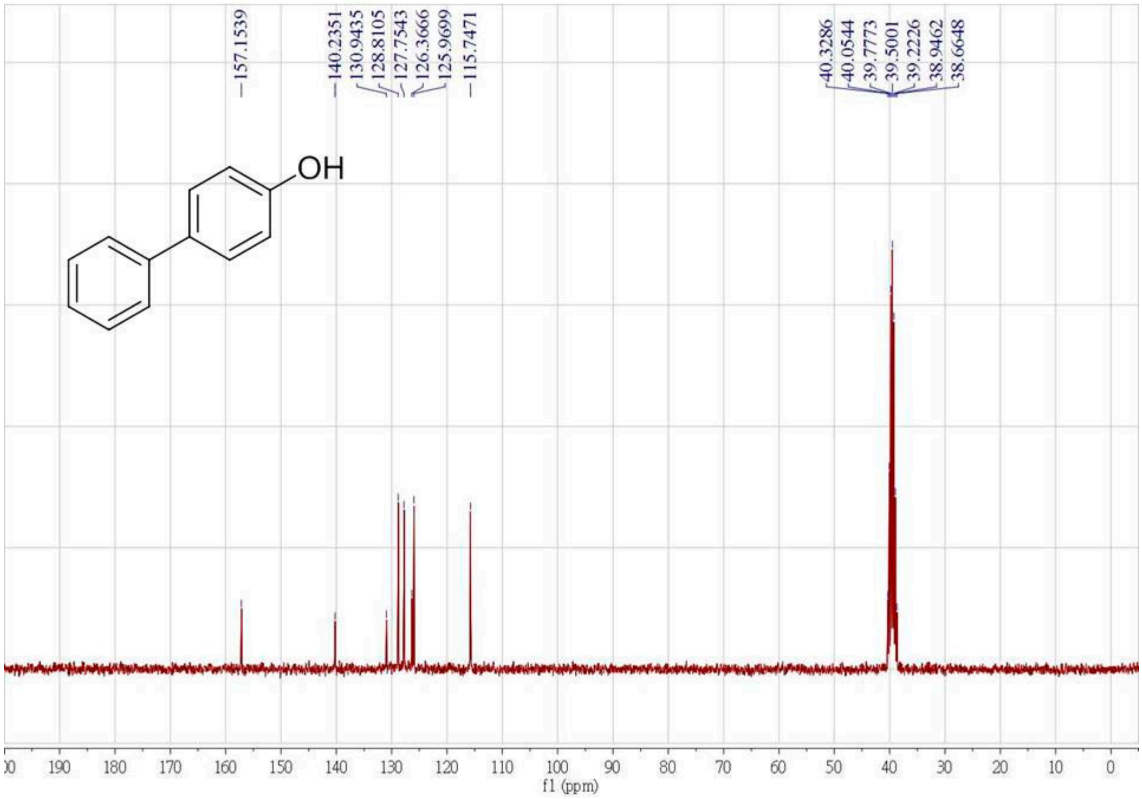

Figure S30. ^{13}C-NMR of compound **3fa**.

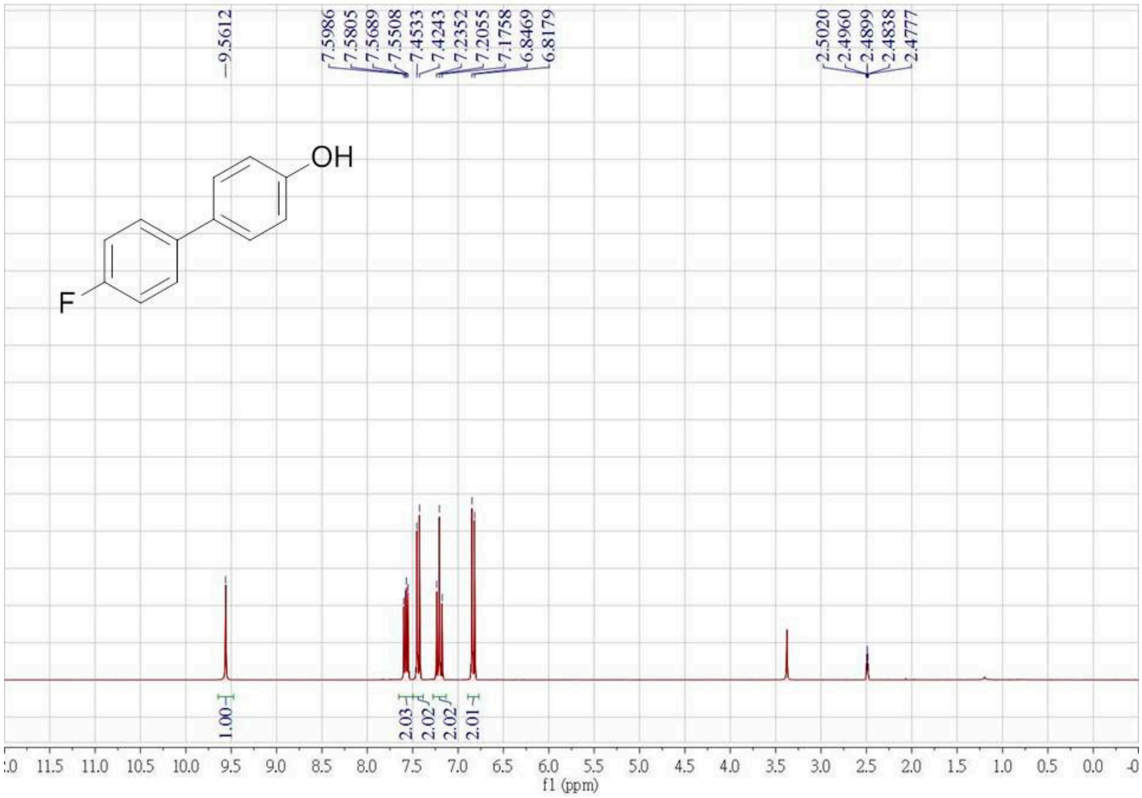

Figure S31. ^{1}H-NMR of compound **3fb**.

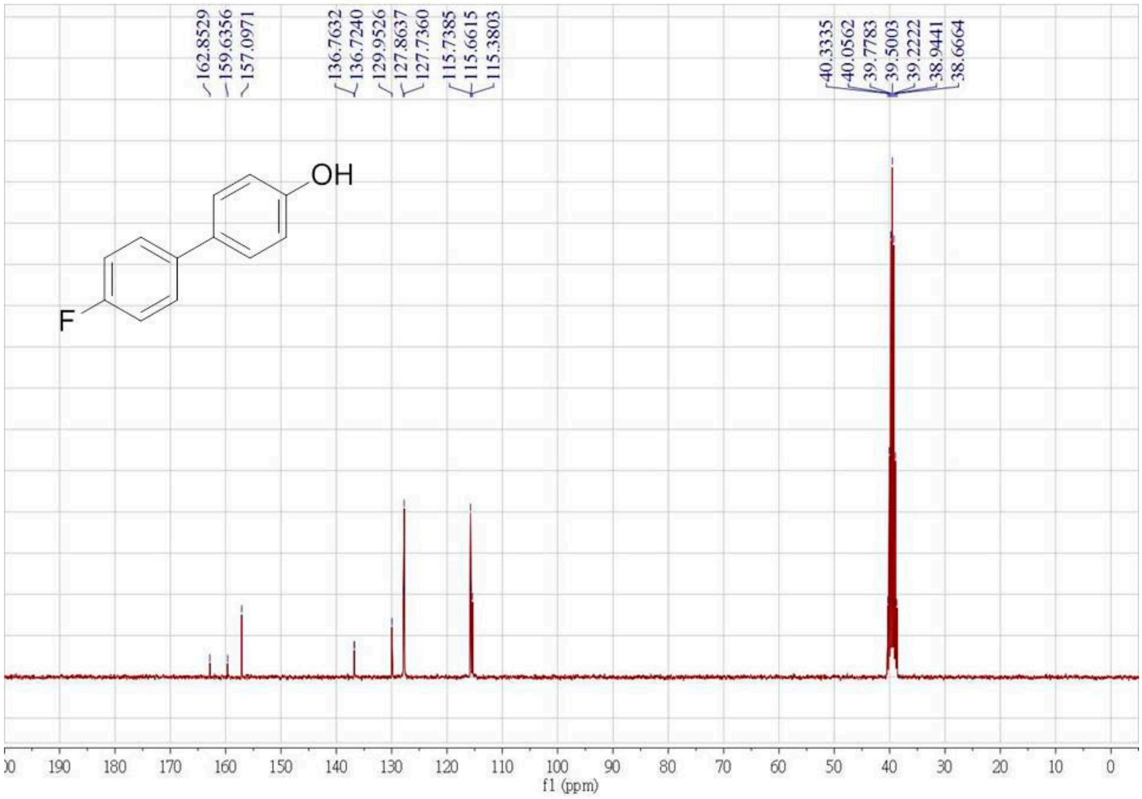

Figure S32. ^{13}C-NMR of compound **3fb**.

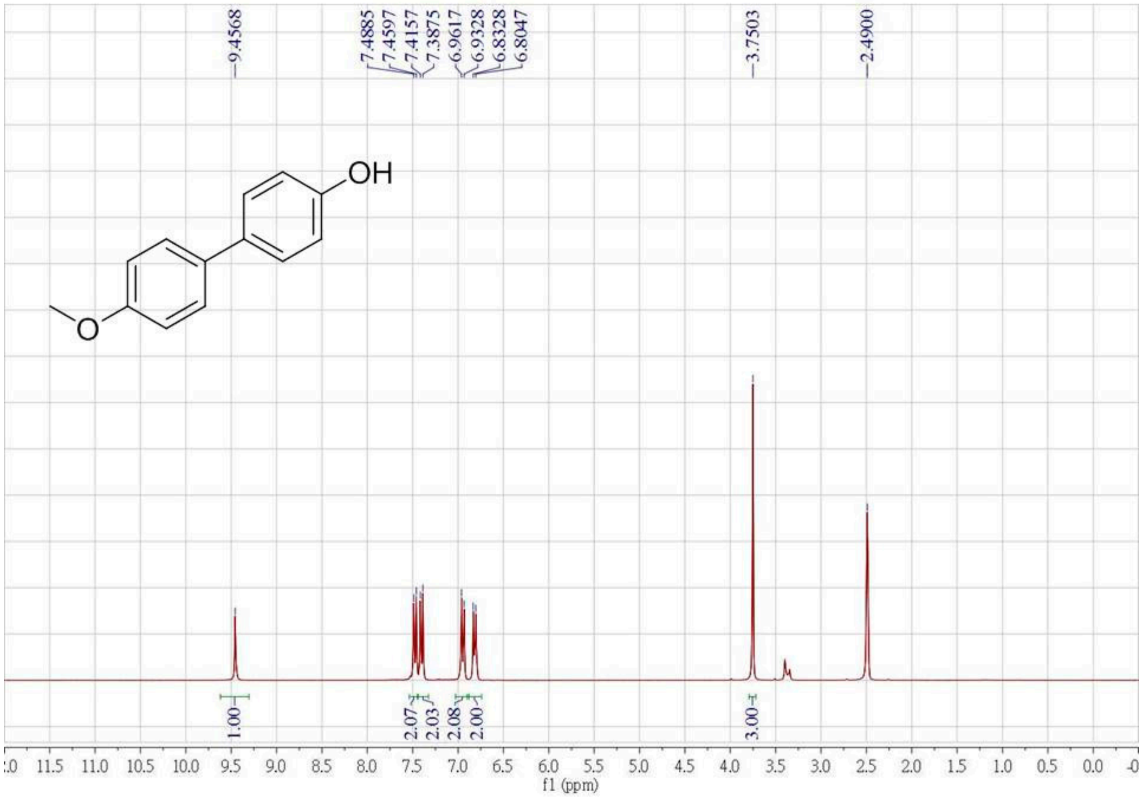

Figure S33. ^{1}H-NMR of compound **3fc**.

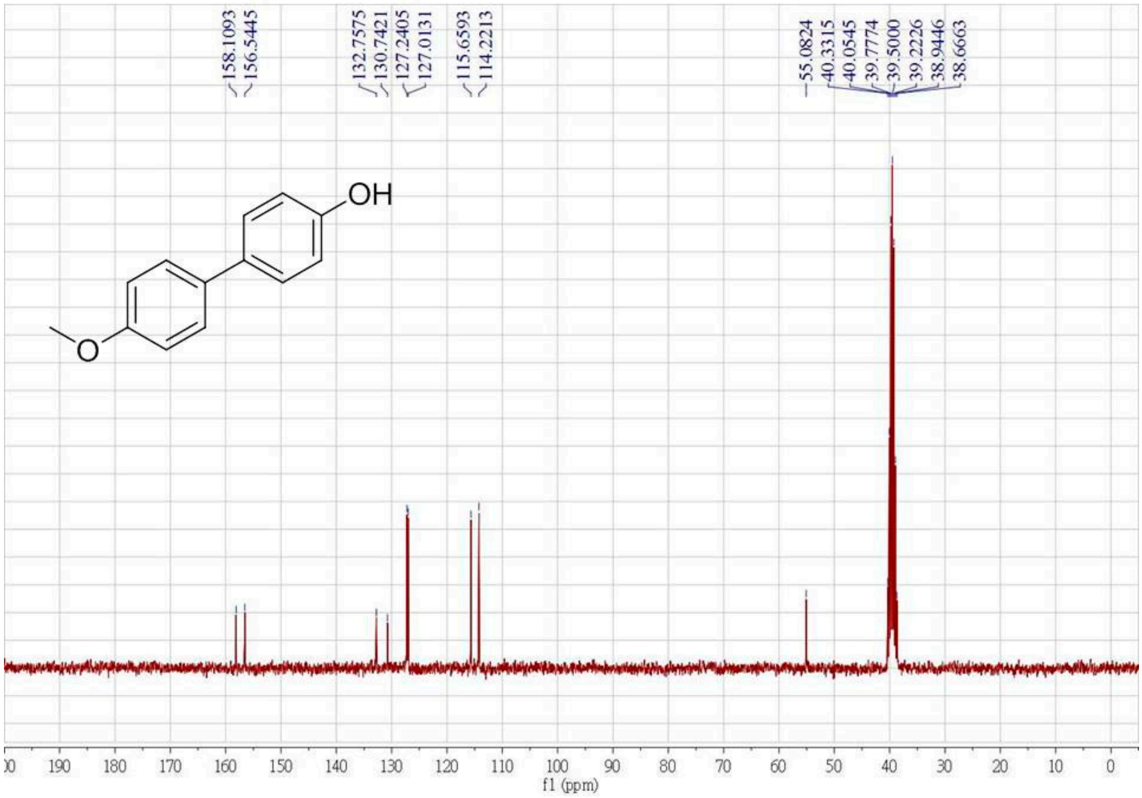

Figure S34. ^{13}C-NMR of compound **3fc**.

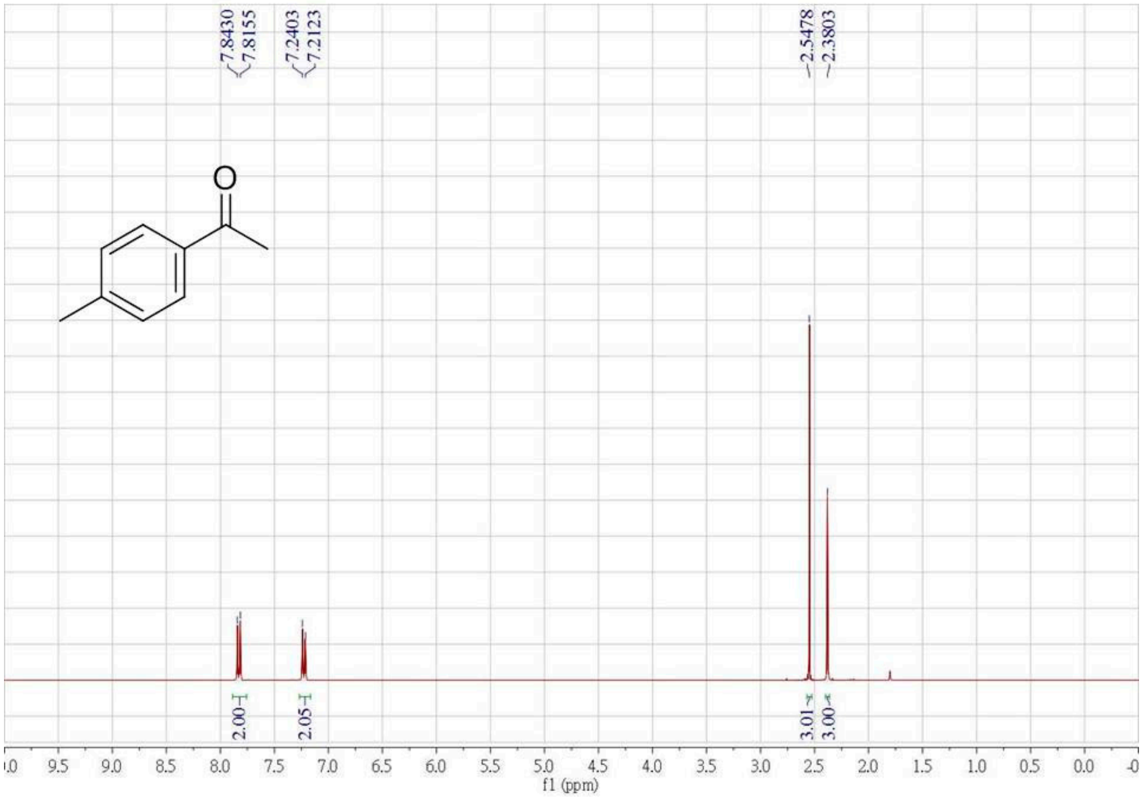

Figure S35. ^{1}H-NMR of compound **6a**.

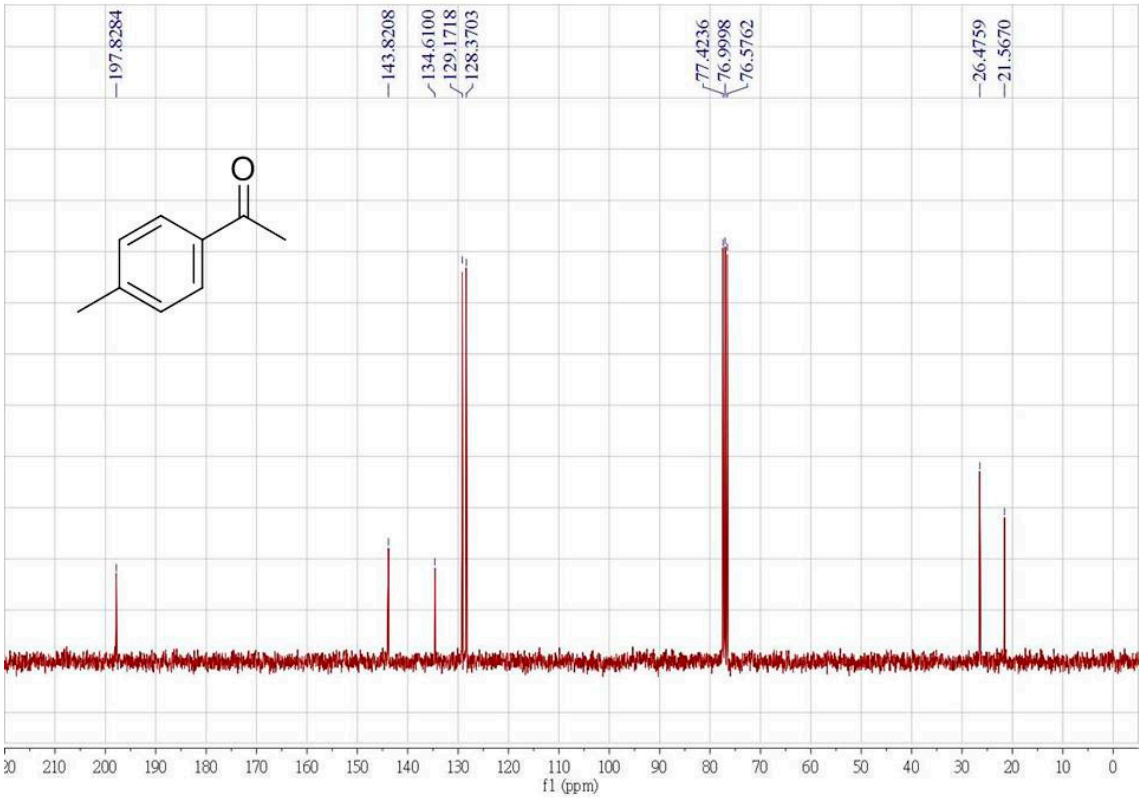

Figure S36. ^{13}C-NMR of compound **6a**.

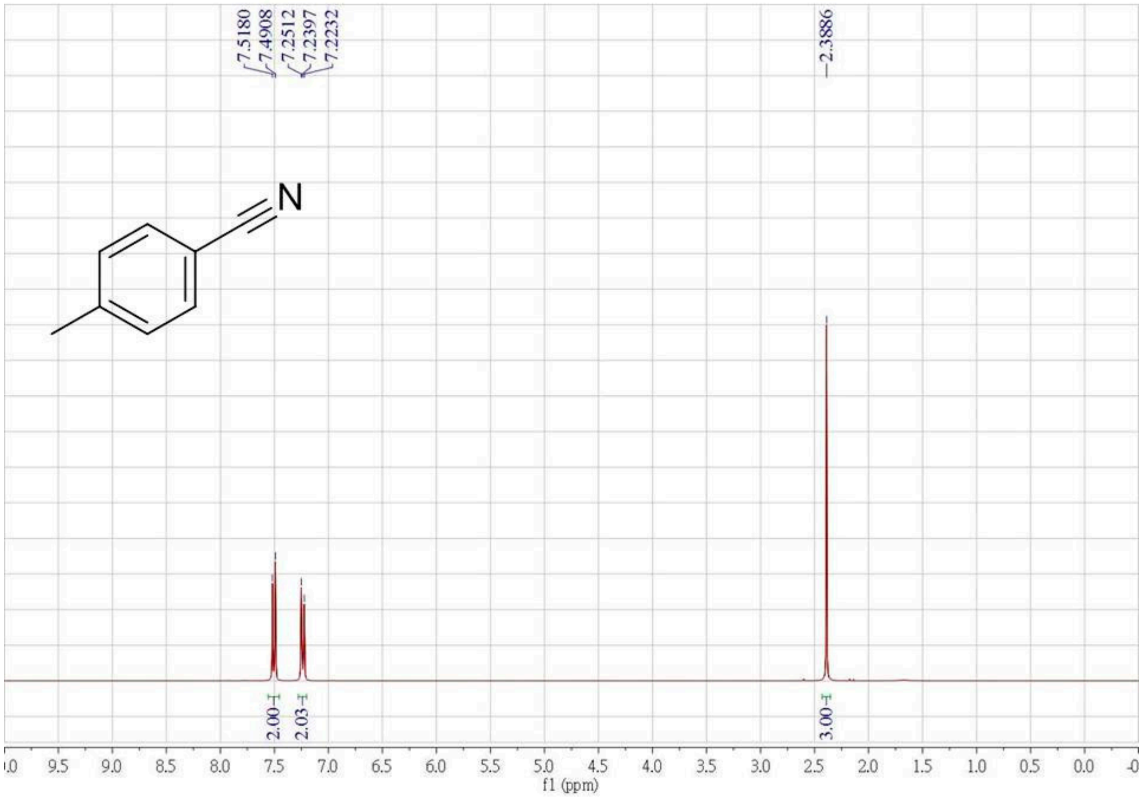

Figure S37. ^{1}H-NMR of compound **6b**.

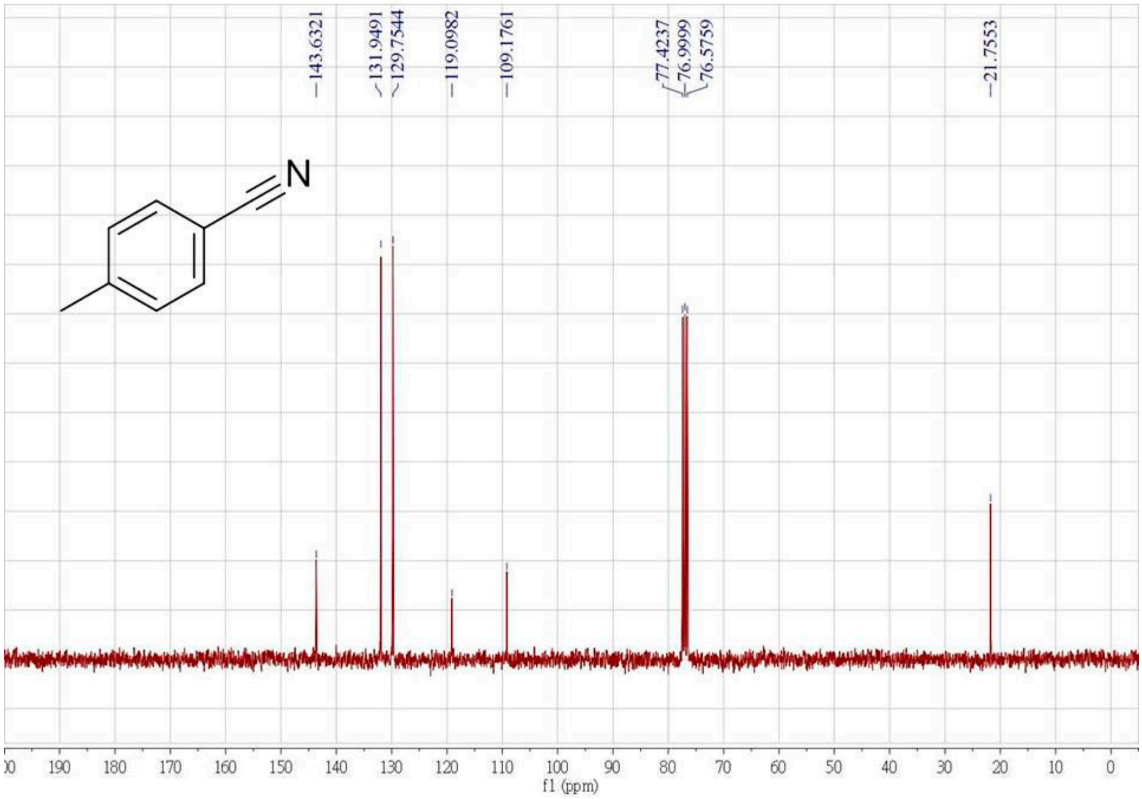

Figure S38. ^{13}C-NMR of compound **6b**.

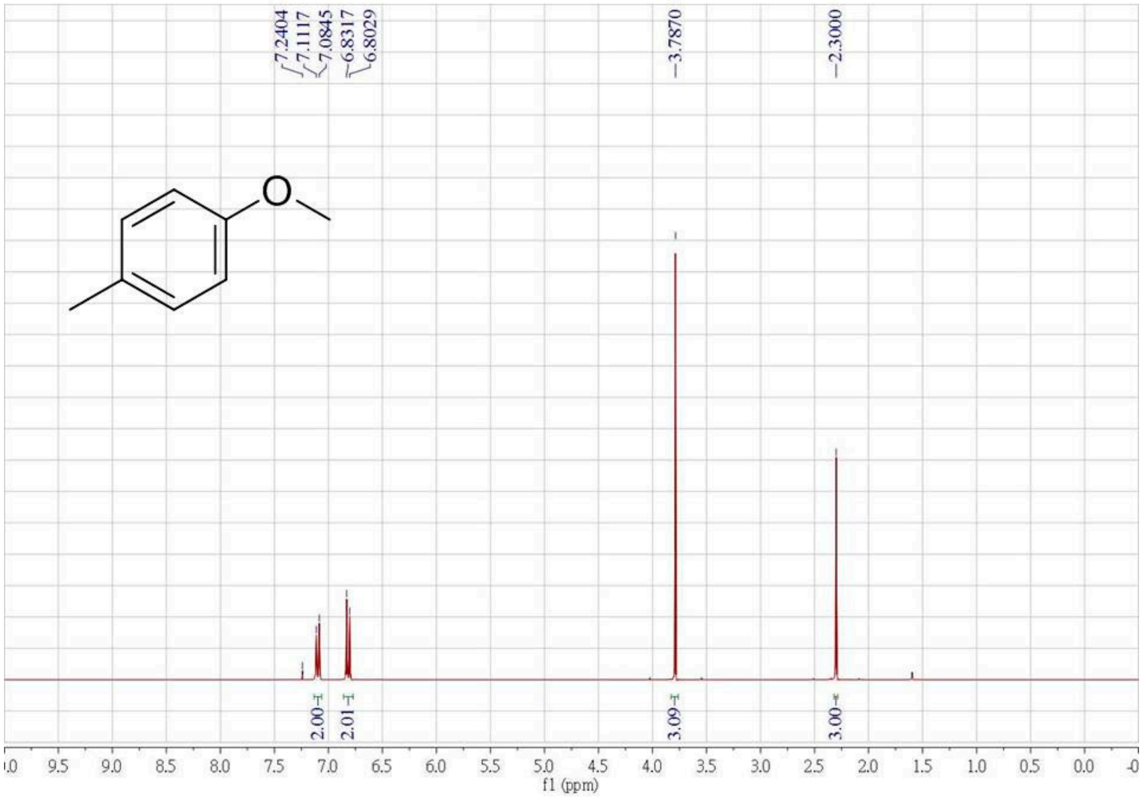

Figure S39. ^{1}H-NMR of compound **6e**.

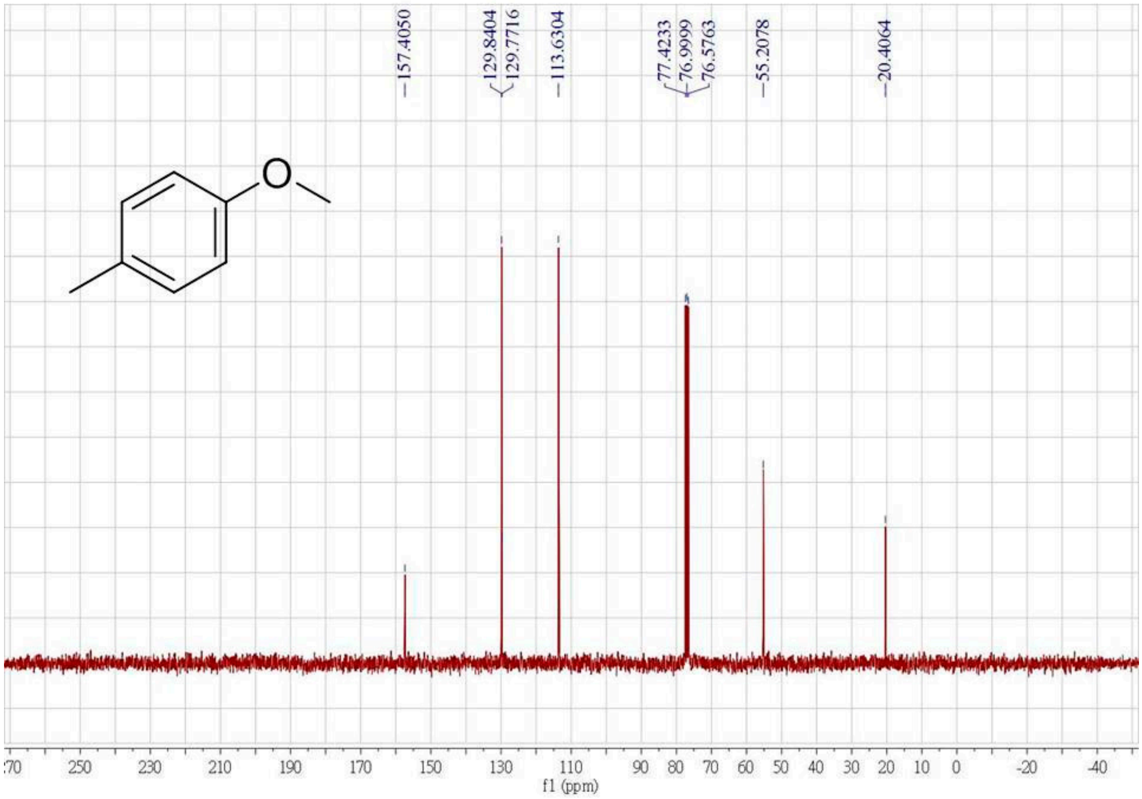

Figure S40. ^{13}C-NMR of compound **6e**.
